# A multi-stage group decision making approach for sustainable supplier selection based on probabilistic linguistic time-ordered incentive operator

**DOI:** 10.1371/journal.pone.0293019

**Published:** 2023-10-31

**Authors:** Faming Zhang, Meixing Li, Zhaoqing Ye, Yufei Niu

**Affiliations:** 1 Business School, Guilin University of Electronic Technology, Guilin, China; 2 College of Foreign Studies, Guilin University of Electronic Technology, Guilin, China; 3 School of Management and Economics, North China University of Water Resources and Electric Power, Zhengzhou, China; National Kaohsiung University of Science and Technology / Industrial University of Ho Chi Minh, TAIWAN

## Abstract

This study proposes a novel multi-stage multi-attribute group decision making method under a probabilistic linguistic environment considering the development state and trend of alternatives. First, the probabilistic linguistic term set (PLTS) is used by decision makers (DMs) to describe qualitative evaluation information. Subsequently, the weights of DMs for different attributes in different periods are determined by the credibility degree, which is combined with the hesitancy degree and the similarity degree. The evaluations of different DMs for alternatives and the evaluations of DMs’ intentions to reward or punish are then aggregated. Later, the trend change level and the trend change stability of alternatives are measured through the means of reward and punishment incentives. Additionally, the probabilistic linguistic time-ordered incentive operator is proposed to aggregate the development state evaluation information and development trend evaluation information in different periods, and alternatives are prioritized by the extended TOPSIS method in the probabilistic linguistic environment. Finally, the practical use of the proposed decision framework is validated by using a sustainable supplier selection problem, and the effectiveness and the applicability of the framework are discussed through comparative analysis. The results show that the proposed approach can select suitable sustainable suppliers by considering their development state and trend in multiple stages.

## Introduction

In recent years, due to natural resource depletion, environmental pollution, labour safety, labour rights and other issues, more and more enterprises are attaching importance to sustainable supply chain management [1, 2]. Sustainable supply chain management involves integrating and achieving a company’s economic, environmental, and social goals by coordinating key business processes to improve the company’s long-term economic performance [3]. Within the realm of sustainable supply chain management, sustainable supplier selection plays a crucial role. The economic performance, as well as environmental and social responsibility performance of upstream suppliers will directly or indirectly influence the overall benefits of downstream enterprises [4]. Therefore, sustainable supplier selection has become a critical decision-making activity in supply chain management [5]. The process of sustainable supplier selection usually involves the participation of multiple relevant departments such as the procurement department, production department, and quality control department. Hence, sustainable supplier selection should be regarded as an extension of the multi-attribute group decision making (MAGDM) problem, taking into account a set of sustainable evaluation criteria and the varying preferences of decision makers (DMs) [6, 7].

In the process of sustainable supplier selection, uncertainty poses a significant challenge due to the subjective, vague, and imprecise nature of judgments on evaluation criteria by DMs [8]. To tackle this issue, Zadeh proposed the concept of traditional fuzzy set (TFS) [9]. Subsequently, various extended forms of TFS gradually gained attention and research, such as the interval-valued fuzzy set (IVFS) [10], the intuitionistic fuzzy set (IFS) [11], the hesitant fuzzy set (HFS) [12], and the Pythagorean fuzzy set (PFS) [13]. However, these fuzzy information types can only quantitatively express the evaluation of sustainable suppliers, and cannot qualitatively describe the uncertainty in DMs’ evaluation opinions. To address this, Zadeh introduced the concept of linguistic term set (LTS) for qualitative evaluation [14]. Rodriguez proposed the concept of a hesitant fuzzy linguistic term set (HFLTS), which simultaneously utilizes multiple linguistic terms to describe complex linguistic information, and provides a more accurate expression of real language evaluation [15]. However, in practical decision-making processes, DMs often have preferences for certain linguistic terms, which may have different levels of importance. HFLTS assigns equal weight to all linguistic terms, which may lead to information loss. Consequently, Pang proposed the concept of probabilistic linguistic term set (PLTS), which considers DMs’ preferences for different linguistic terms by assigning different probabilities to them [16]. The rationality and flexibility of PLTS have attracted increasing attention from scholars, who have studied PLTS from various perspectives, including its operational laws [17, 18], integration with MAGDM [19, 20], and applications of probabilistic linguistic preference relations [21–23], etc. In addition, PLTS has found wide applications in fields such as patients prioritization assessment [24], evaluation and selection of online learning platforms [25], and risk assessment of marine ranching equity financing [26]. However, there is a lack of research that applies PLTS to the problem of sustainable supplier selection. In fact, sustainable supplier selection often involves a lot of fuzzy and uncertain linguistic decision information, and PLTS can effectively meet the needs of evaluation and decision in this process.

In the study of sustainable supplier selection, aggregating DMs is a crucial step, and determining the weights of DMs is key to this process. Grošelj proposed an improved symmetric projection method to calculate the weights of DMs in the AHP process [27]. Davoudabadi and Mohagheghi considered both subjective and objective weights of DMs comprehensively [28, 29]. Liu and Meng determined the weights of DMs based on the similarity degree of their evaluations [30, 31]. Li calculated the weights of DMs by considering both the similarity and uncertainty of their evaluations [32]. However, in the aforementioned research methods, each DM is assigned the same weight for different attributes, in reality, each DM specializes in different areas, and typically has expertise in specific domains rather than all areas. In addition, DMs has varying levels of knowledge that may change over time. Therefore, it is more realistic and reasonable to assign different weights to DMs for different attributes and periods.

In recent years, research on the MAGDM problem of sustainable supplier selection has also yielded fruitful results. The most widely used methods to solve the problem of sustainable supplier selection include Analysis Network Procedures (ANP) method [33, 34], Data Envelopment Analysis (DEA) [35–37], Decision Making Trial and Evaluation Laboratory (DEMATEL) [38–40], Technique for Order Preference by Similarity to an Ideal Solution (TOPSIS) [41–43], VlseKriterijuska Optimizacija I Komoromisno Resenje (VIKOR) method [44–46], the best-worst method (BWM) [47–49], PROMETHEE [50, 51] and multi-objective optimization methodology [52, 53]. Most of the above studies only consider the decision information of a single period for sustainable supplier selection. However, the sustainable development of suppliers in the economic, environmental, and social dimension is a long-term process, and sustainable suppliers’ comprehensive performance in multiple periods is crucial, but rarely mentioned. Olanrewaju proposed a multi-stage stochastic programming model to solve the supplier selection problem in disaster response [54]. Kaur proposed a multi-stage hybrid model for integrated supplier segmentation, selection and order allocation [55]. Guo proposed a multi-stage multi-attribute group decision making method that considers the psychological state of DMs in the group decision making process [56]. Li proposed a group decision-making approach for supplier selection by analyzing the influence of time factors and opinion interaction between DMs [57]. Xie proposed a dynamic group DEMATEL decision-making method involving multiple stages, factors and experts complex decision-making situations [58]. The above researches mainly focuses on examining the development state of suppliers through evaluation values within different periods. However, in the actual sustainable supplier selection process, DMs often expect sustainable suppliers to achieve both rapid and stable development, but the research on the development trend of sustainable suppliers is still relatively insufficient.

To make up for the shortcomings of previous researches, the research motivations of this study are as follows: (1) In the face of increasingly complex decision-making environment, it is particularly important to obtain efficiently evaluation information. The PLTS can more effectively handle the uncertainty of DMs and more fully express their preferences. (2) Due to the fact that most existing methods for determining DMs’ weights do not consider the differences and variability of DMs’ attribute cognition, this study determines DMs’ weights for different attributes at different stages based on their credibility. (3) To further evaluate the development trend of sustainable suppliers, the means of reward and punishment incentives is used to explore the differences in the development trend of alternatives, and the PLTOI operator is proposed to aggregate the development trend decision information. (4) Sustainable supplier selection is a very important part of supply chain management. Scientific and reasonable selection of sustainable suppliers has a far-reaching positive impact on improving sustainable supply chain management. (5) The method proposed in this paper can effectively solve the problems of the multi-stage multi-attribute group decision which decision-making information is expressed by PLTSs.

Therefore, this paper proposes a new method for multi-stage multi-attribute group decision making to select sustainable suppliers. The approach consists of several steps. Firstly, DMs utilize the PLTS to qualitatively express their evaluations of sustainable suppliers for various attributes and their own reward-punishment intentions. Secondly, the weights of DMs are determined based on their credibility for different attributes in different periods. These weights are then used to aggregate DMs’ evaluations of sustainable suppliers and their evaluations of reward-punishment intentions. Thirdly, the development trend of sustainable suppliers is assessed according to DMs’ subjective reward-punishment preferences, and the development state evaluations and the development trend evaluations in different periods are aggregated using the aggregation operator called PLTOI, then the extended TOPSIS method is employed in the probabilistic linguistic environment to rank sustainable suppliers. Finally, the effectiveness and applicability of the proposed method are demonstrated through a case study involving a civil UAV manufacturing enterprise.

The main innovations of this paper are as follows: (1) The use of PLTS to represent DMs’ decision information, which effectively handles the uncertainty in the evaluation of sustainable supplier selection. (2) The assignment of weights to DMs for different attributes in different periods, takes into account the hesitancy and similarity of their evaluations, based on the characteristics of PLTS. (3) Consideration of the differences in the development trends of alternatives and obtaining relevant decision information through reward incentives and punishment incentives. (4) Introduction of the probabilistic linguistic time-ordered incentive (PLTOI) operator to summarize the development state evaluations and development trend evaluations of alternatives in different periods.

The rest of this paper is structured as follows: In Section 2, the concepts of PLTS and the extended TOPSIS method in the probabilistic linguistic environment are reviewed briefly. Section 3 presents a multi-stage group decision making method based on the probabilistic linguistic time-ordered incentive operator to address the issue of sustainable supplier selection. An example of sustainable supplier selection to validate the proposed approach is presented in Section 4. Finally, Section 5 concludes the paper and discusses future work.

## Preliminaries

This section introduces some fundamental concepts associated with PLTS and the extended TOPSIS method using probabilistic linguistic information.

### PLTS

**Definition 1** [16]. Let $S=\{s_{\alpha }|\alpha =-\tau ,\cdots ,0,\cdots ,\tau \}$ be a LTS, a PLTS can be defined as:

$$L(p)=\{L^{\left(l\right)}(p^{\left(l\right)})|L^{\left(l\right)}\in S,)r^{\left(l\right)}\in \alpha ,p^{\left(l\right)}\geq 0,l=1,2,\cdots ,\#L,\sum_{l=1}^{\#L}p^{\left(l\right)}\leq 1\}$$ (1)

where *L*^(*l*)^(*p*^(*l*)^) is the linguistic term *L*^(*l*)^ associated with the probability *p*^(*l*)^, *r*^(*l*)^ is the subscript of the linguistic term *L*^(*l*)^, and #*L* is the number of all different linguistic terms in *L*(*p*).

**Definition 2** [16]. Given a PLTS *L*(*p*) with $\sum_{l=1}^{\#L}p^{\left(l\right)}<1$, then the associated PLTS $\hat{L}(p)$ is defined by:

$$\hat{L}(p)=\{L^{\left(l\right)}({\hat{p}}^{\left(l\right)})|L^{\left(l\right)}\in S,)r^{\left(l\right)}\in \alpha ,{\hat{p}}^{\left(l\right)}\geq 0,l=1,2,\cdots ,\#L\}$$ (2)

where ${\hat{p}}^{\left(l\right)}=p^{\left(l\right)}/\sum_{l=1}^{\#L}p^{\left(l\right)}$, for all *l* = 1,2,⋯,#*L*.

**Definition 3** [16]. Let *L*_1_(*p*) and *L*_2_(*p*) be any two PLTSs, $L_{1}(p)=\{L_{1}^{\left(l\right)}(p_{1}^{\left(l\right)})|l=1,2,\cdots \#L_{1}\}$ and $L_{2}(p)=\{L_{2}^{\left(l\right)}(p_{2}^{\left(l\right)})|l=1,2,\cdots ,\#L_{2}\}$, and let #*L*_1_ and #*L*_2_ be the numbers of linguistic terms in *L*_1_(*p*) and *L*_2_(*p*) respectively. If #*L*_1_≻#*L*_2_, then we will add #*L*_1_−#*L*_2_ linguistic terms to *L*_2_(*p*) so that the numbers of linguistic terms in *L*_1_(*p*) and *L*_2_(*p*) are identical. The added linguistic terms are the smallest ones in *L*_2_(*p*), and the probabilities of all the linguistic terms are zero.

Let $L_{1}(p)=\{L_{1}^{\left(l\right)}(p_{1}^{\left(l\right)})|l=1,2,\cdots ,\#L_{1}\}$ and $L_{2}(p)=\{L_{2}^{\left(l\right)}(p_{2}^{\left(l\right)})|l=1,2,\cdots ,\#L_{2}\}$, then the normalization process can be conducted by the following two steps:

1. If $\sum_{l=1}^{\#L}p^{\left(l\right)}<1$, then by the Formula (2), we calculate ${\hat{L}}_{i}(p)(i=1,2)$.
2. If #*L*_1_≠#*L*_2_, then according to Definition 3, we add some elements to the one with the smaller number of elements.

The resultant PLTSs are called the normalized PLTSs. For the convenience of presentation, the normalized PLTSs are denoted by *L*_1_(*p*) and *L*_2_(*p*) as well.

**Definition 4** [16]. Let $L(p)=\{L^{\left(l\right)}(p^{\left(l\right)})|L^{\left(l\right)}\in S,p^{\left(l\right)}\geq 0,l=1,2,\cdots ,\#L\}$ be a PLTS, and *r*^(*l*)^ be the subscript of linguistic term *L*^(*l*)^. Then the score of L(p) is:

$$E(L(p))=s_{\bar{\alpha }}$$ (3)

where $\bar{\alpha }=\sum_{l=1}^{\#L}r^{\left(l\right)}p^{\left(l\right)}/\sum_{l=1}^{\#L}p^{\left(l\right)}$.

**Definition 5** [17]. Let $L_{i}(p)=\{L_{i}^{\left(l\right)}(p_{i}^{\left(l\right)})|l=1,2,\cdots ,\#L_{i}\}(i=1,2)$ be two standardized PLTSs, and *λ* be a positive real number, $\eta_{i}^{\left(l\right)}\in g(L_{i})(l=1,2,\cdots ,\#L_{i})$. Then the basic operations of PLTSs are as follows:

$$L_{1}(p)+L_{2}(p)=g^{-1}(\underset{\eta_{1}^{\left(l\right)}\in g(L_{1}),\eta_{2}^{\left(l\right)}\in g(L_{2})}{\cup }\left\{\left(\eta_{1}^{\left(l\right)}+\eta_{2}^{\left(l\right)}-\eta_{1}^{\left(l\right)}\eta_{2}^{\left(l\right)})(p_{1}^{\left(l\right)}p_{2}^{\left(l\right)}\right)\right\})$$ (4)

$$\lambda L_{i}(p)=g^{-1}(\underset{\eta_{i}^{\left(l\right)}\in g(L_{i})}{\cup }\left\{\left(1-{\left(1-\eta_{i}^{\left(l\right)}\right)}^{\lambda })(p_{i}^{\left(l\right)}\right)\right\})$$ (5)

**Definition 6** [16]. Let $L_{i}(p)=\{L_{i}^{\left(l\right)}(p_{i}^{\left(l\right)})|l=1,2,\cdots ,\#L_{i})\}(i=1,2,\cdots ,n)$ be *n* standardized PLTSs, where $L_{i}^{\left(l\right)}$ and $p_{i}^{\left(l\right)}$ are the *l*th linguistic term and its probability respectively in *L*_*i*_(*p*). Let *ω* = (*ω*_1_,*ω*_2_,⋯,*ω*_*n*_)^*T*^ is the weight vector of *L*_*i*_(*p*)(*i* = 1, 2,⋯,*n*), *ω*_*i*_≥0(*i* = 1,2,⋯,*n*), and $\sum_{i=1}^{n}\omega_{i}=1$. Then the probabilistic linguistic weighted averaging (PLWA) operator is as follows:

$$\mathrm{PLWA}(L_{1}(p),L_{2}(p),\cdots ,L_{n}(p))=\overset{n}{\underset{i=1}{⊕}}\omega_{i}L_{i}(p)$$ (6)

**Definition 7** [16]. Let $L_{i}(p)=\{L_{i}^{\left(l\right)}(p_{i}^{\left(l\right)})|l=1,2,\cdots ,\#L_{i}\}(i=1,2)$ be two standardized PLTSs, then the deviation degree between *L*_1_(*p*) and *L*_2_(*p*) is as follows:

$$d(L_{1}(p),L_{2}(p))=\sqrt{\frac{1}{\#L_{1}}\sum_{l=1}^{\#L_{1}}{\left(p_{1}^{\left(l\right)}r_{1}^{\left(l\right)}-p_{2}^{\left(l\right)}r_{2}^{\left(l\right)}\right)}^{2}}$$ (7)

### The extended TOPSIS method with probabilistic linguistic information

TOPSIS was proposed by Hwang and Yoon to rank and select alternatives by calculating geometric distance [59]. This method does not impose strict restrictions on data distribution or the number of attributes, and can effectively calculate the comprehensive impact of multiple attributes. Consequently, TOPSIS is widely used in various fuzzy uncertain environments [60, 61]. Pang proposed the extended TOPSIS method with probabilistic linguistic information, which follows the process outlined below [16]:

Let *O* = {*o*_1_,*o*_2_,⋯,*o*_*n*_}(*n*≥2,*i* = 1,2,⋯,*n*) be the alternatives and *X* = {*x*_1_,*x*_2_,⋯,*x*_*j*_}

((*j*≥2, *j* = 1,2,⋯,*m*) be the evaluation attributes. Let *L* = [*L*_*ij*_(*p*)]_*n*×*m*_ be a probabilistic linguistic decision matrix. Then $L_{ij}(p)=\{L_{ij}^{\left(l\right)}(p_{ij}^{\left(l\right)})|l=1,2,\cdots ,\#L_{ij}\}$ is defined as the vector of the attribute *x*_*j*_ of the alternative *o*_*i*_.

#### Step 1

Determine the positive ideal solution *L*^+^(*p*) and the negative ideal solution *L*^−^(*p*):

$L{\left(p\right)}^{+}=(L_{1}{\left(p\right)}^{+},L_{2}{\left(p\right)}^{+},\cdots ,L_{m}{\left(p\right)}^{+})$, where $L_{j}{\left(p\right)}^{+}=\{{\left(L_{j}^{\left(l\right)}\right)}^{+}|l=1,2,\cdots ,\#L_{ij}\}$ $=\{\{{\left(L_{j}^{\left(1\right)}\right)}^{+},{\left(L_{j}^{\left(2\right)}\right)}^{+},\cdots ,{\left(L_{j}^{\left(\#L_{ij}\right)}\right)}^{+}\}$, it’s calculated by the following formula:

$${\left(L_{j}^{\left(l\right)}\right)}^{+}=s_{\underset{i}{\max}\{p_{ij}^{\left(l\right)}r_{ij}^{\left(l\right)}\}},l=1,2,\cdots ,\#L_{ij},j=1,2,\cdots ,m$$ (8)

where $r_{ij}^{\left(l\right)}$ is the subscript of the linguistic term $L_{ij}^{\left(l\right)}$.

$L{\left(p\right)}^{-}=(L_{1}{\left(p\right)}^{-},L_{2}{\left(p\right)}^{-},\cdots ,L_{m}{\left(p\right)}^{-})$, where $L_{j}{\left(p\right)}^{-}=\{{\left(L_{j}^{\left(l\right)}\right)}^{-}|l=1,2,\cdots ,\#L_{ij}\}$ $=\{{\left(L_{j}^{\left(1\right)}\right)}^{-},){(L_{j}^{\left(2\right)}}^{-},\cdots ,{\left(L_{j}^{\left(\#L_{ij}\right)}\right)}^{-}$, it’s calculated by the following formula:

$${\left(L_{j}^{\left(l\right)}\right)}^{-}=s_{\underset{i}{\min}\{p_{ij}^{\left(l\right)}r_{ij}^{\left(l\right)}\}},l=1,2,\cdots ,\#L_{ij},j=1,2,\cdots ,m$$ (9)

where $r_{ij}^{\left(l\right)}$ is the subscript of the linguistic term $L_{ij}^{\left(l\right)}$.

#### Step 2

Calculate the deviation degree between the alternative and the positive ideal solution, and the deviation degree between the alternative and the negative ideal solution:

$$d(o_{i},L{\left(p\right)}^{+})=\sum_{j=1}^{m}d(L_{ij}(p),L_{j}{\left(p\right)}^{+})=\sum_{j=1}^{m}\sqrt{\frac{1}{\#L_{ij}}\sum_{l=1}^{\#L_{ij}}{\left(p_{ij}^{\left(l\right)}r_{ij}^{\left(l\right)}-{\left(p_{j}^{\left(l\right)}r_{j}^{\left(l\right)}\right)}^{+}\right)}^{2}}$$ (10)

$$d(o_{i},L{\left(p\right)}^{-})=\sum_{j=1}^{m}d(L_{ij}(p),L_{j}{\left(p\right)}^{-})=\sum_{j=1}^{m}\sqrt{\frac{1}{\#L_{ij}}\sum_{l=1}^{\#L_{ij}}{\left(p_{ij}^{\left(l\right)}r_{ij}^{\left(l\right)}-{\left(p_{j}^{\left(l\right)}r_{j}^{\left(l\right)}\right)}^{-}\right)}^{2}}$$ (11)

The smaller the distance *d*(*o*_*i*_,*L*(*p*)^+^) implies, the better the alternative *o*_*i*_, and the larger the distance *d*(*o*_*i*_,*L*(*p*)^+^) implies, the better the alternative *o*_*i*_. Thus, we let $d_{\min}(o_{i},L{\left(p\right)}^{+})=\underset{1\leq i\leq n}{\min}d_{\max}(o_{i},L{\left(p\right)}^{+})$ be the smallest deviation degree between the alternative *o*_*i*_ and the positive ideal solution, and let $d_{\max}(o_{i},L{\left(p\right)}^{-})=\underset{1\leq i\leq n}{\max}d_{\max}(o_{i},L{\left(p\right)}^{-})$ be the largest deviation degree between the alternative *o*_*i*_ and the negative ideal solution.

#### Step 3

Calculate the closeness coefficient to the ideal solutions:

$$CI(o_{i})=\frac{d(o_{i},L{\left(p\right)}^{-})}{d_{\max}(o_{i},L{\left(p\right)}^{-})}-\frac{d(o_{i},L{\left(p\right)}^{+})}{d_{\min}(o_{i},L{\left(p\right)}^{-})}$$ (12)

where *CI*(*o*_*i*_)≤0(*i* = 1,2,⋯,*n*), the larger the closeness coefficient *CI*(*o*_*i*_), the better the alternative *o*_*i*_.

#### Step 4

Rank the preference order and select the best alternative.

Rank the order according to *CI*(*o*_*i*_) ascending order.

## The multi-stage group decision making approach based on the PLTOI operator

**Fig 1:**
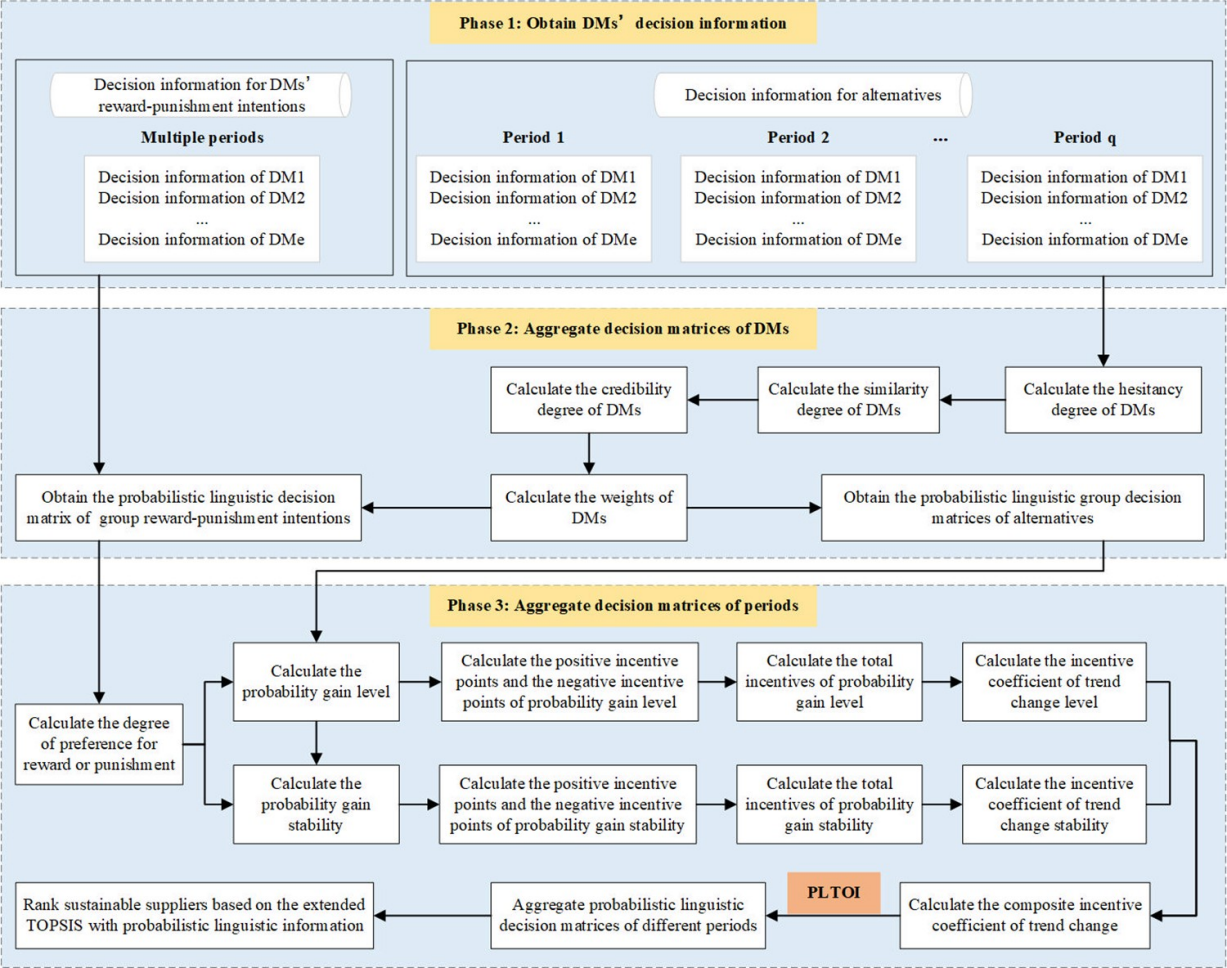
Flowchart of the proposed approach for sustainable supplier selection problem.

In this section, we propose a novel multi-stage group decision making approach based on the PLTOI operator for selecting sustainable suppliers. Let *C* = {*c*_1_,*c*_2_,⋯,*c*_*e*_} be a set of DMs, *O* = {*o*_1_,*o*_2_,⋯,*o*_*n*_} be a set of alternative sustainable suppliers, *X* = {*x*_1_,*x*_2_,⋯,*x*_*m*_} be a set of evaluation attributes, and *T* = {*t*_1_,*t*_2_,⋯*t*_*q*_} be a set of periods. The linguistic term set for sustainable suppliers’ performance is $S=\{s_{r}|r=-\tau ,\cdots ,0,\cdots ,\tau \}$, where *s*_*r*_(0<*r*≤*τ*) are pre-set as the language terms indicating the degree of good performance, *s*_*r*_(−*τ*≤*r*<0) are pre-set as the language terms indicating the degree of poor performance, and *s*_0_ is pre-set as a language term indicating medium performance. The linguistic term set for DMs’ reward-punishment intentions is $\tilde{S}=\{{\tilde{s}}_{\tilde{r}}|\tilde{r}=-\tau ,\cdots ,0,\cdots ,\tau \}$, where ${\tilde{s}}_{\tilde{r}}(0<\tilde{r}\leq \tau )$ are pre-set as language terms indicating the degree of preference for reward, ${\tilde{s}}_{\tilde{r}}(-\tau \leq \tilde{r}<0)$ are pre-set as language terms indicating the degree of preference for punishment, and ${\tilde{s}}_{0}$is pre-set as a language term indicating neither preference for reward nor punishment. The basic procedure of the proposed approach is shown in Fig 1 and the detailed steps of the approach are described as follows.

### Aggregate the probabilistic linguistic evaluations of different DMs

#### Step 1

Obtain the decision matrices.

Obtain the probabilistic linguistic evaluations for sustainable suppliers and the probabilistic linguistic evaluations for DMs’ own reward-punishment intentions. ${\bar{L}}_{gk}={\left[{\bar{L}}_{ij}^{{}_{gk}}(\bar{p})\right]}_{n\times m}$ is the decision matrix given by *c*_*g*_ in period *t*_*k*_ based on the LTS $S=\{s_{r}|r=-\tau ,\cdots ,0,\cdots ,\tau \}$, where ${\bar{L}}_{ij}^{{}_{gk}}(p)=\{{\bar{L}}_{ij,gk}^{\left(l\right)}({\bar{p}}_{ij,gk}^{\left(l\right)})|{\bar{L}}_{ij,gk}^{\left(l\right)}\in S,\bar{l}=1,2,\cdots ,\#{\bar{L}}_{ij,gk}\}$ is a PTLS representing the evaluations of *o*_*i*_ for attribute *x*_*j*_. $\tilde{L}={\left[{\tilde{L}}_{g}(\tilde{p})\right]}_{1\times m}$ is the decision matrix given by DMs based on the LTS $\tilde{S}=\{{\tilde{s}}_{\tilde{r}}|\tilde{r}=-\tau ,\cdots ,0,\cdots ,\tau \}$, where ${\tilde{L}}_{g}(\tilde{p})=\{{\tilde{L}}_{g}^{\left(\tilde{l}\right)}({\tilde{p}}_{g}^{\left(\tilde{l}\right)})|{\tilde{L}}_{g}^{\left(\tilde{l}\right)}\in \tilde{S},\tilde{l}=1,2,\cdots ,\#{\tilde{L}}_{g}\}$ is a PTLS representing the evaluations of *c*_*g*_ for his own reward-punishment intentions.

#### Step 2

Standardize the decision matrices.

Standardize the obtained decision matrices. The original matrix ${\bar{L}}_{gk}$ can be standardized to the decision matrix $L_{gk}={\left[L_{ij}^{{}_{gk}}(p)\right]}_{n\times m}$ by the formula as follows [62]:

$$neg(\bar{L}(\bar{p}))=\{L^{\left(l\right)}(p^{\left(l\right)})|L^{\left(l\right)}=neg({\bar{L}}^{\left(\bar{l}\right)})=neg(s_{{\bar{r}}^{\left(\bar{l}\right)}});\bar{l}=1,2,\cdots ,\#\bar{L}\}$$ (13)

where $neg(s_{{\bar{r}}^{\left(\bar{l}\right)}})$ is the negative operation for linguistic terms, defined as *neg*(*s*_*α*_) = *s*_−*α*_, *neg*(*s*_0_) = *s*_0_ in particular.

#### Step 3

Aggregate the probabilistic linguistic evaluations of different DMs.

To address the sustainable supplier selection problem, it is necessary to aggregate the evaluations of different DMs to obtain a group evaluation for each alternative regarding different attributes. However, as DMs may possess varying professional backgrounds, levels of knowledge, experiences, and perceptions that evolve over different periods, their credibility levels can differ when assessing different attributes. The main factors contributing to these credibility differences are DMs’ hesitancy degree and similarity degree. Therefore, in this step, the credibility degree is initially determined, followed by the calculation of DMs’ weights and the aggregation of their evaluations based on this foundation.

**Step 3–1.** Calculate the weights of different DMs.

**Step 3-1-1.** Calculate the hesitancy degree of DMs.

Due to the complexity and uncertainty of the decision-making environment, it is natural for DMs to exhibit a certain level of hesitation when providing evaluations. The degree of hesitation among DMs primarily manifests in terms of the number of linguistic terms used, the extent of deviation between linguistic terms, and the completeness of probability information in probabilistic linguistic evaluations. Specifically, a higher number of linguistic terms, larger deviation between linguistic terms, and lower completeness of probability information contribute to a greater hesitation degree among DMs. For the evaluations of alternatives regarding attribute *x*_*j*_ by *c*_*g*_ in period *t*_*k*_, the hesitancy degree is denoted as $hd_{j}^{gk}$, which can be calculated using the following formula:

$$hd_{j}^{gk}=\frac{1}{m}\sum_{i=1}^{m}\left(\frac{1}{3}(\left(1-\frac{\#L_{ij,gk}-1}{2\tau +1})+P_{ij}^{gk}\times \chi_{ij}^{gk}+(1-P_{ij}^{gk}\right))\right)$$ (14)

where $P_{ij}^{gk}$ represents the total probability value in unstandardized probabilistic linguistic evaluation ${\bar{L}}_{ij}^{gk}(p)$, $\chi_{ij}^{gk}=\frac{1}{\#L_{ij,gk}}\sum_{l=1}^{\#L_{ij,gk}}\left(p_{ij,gk}^{\left(l\right)}{\left(r_{ij,gk}^{\left(l\right)}-{\bar{\alpha }}_{ij,gk}\right)}^{2}\right)$ represents the variance of probabilistic linguistic evaluation $L_{ij}^{gk}(p)$.

**Step 3-1-2.** Calculate the similarity degree of DMs.

The similarity degree of a DM indicates the consistency between the DM and other DMs. The consistency of the DM is determined by the difference between the evaluations of that DM and those of other DMs. The smaller the difference, the higher the consistency of the DM. For the evaluations of alternatives regarding attribute *x*_*j*_ by *c*_*g*_ in period *t*_*k*_, the similarity degree is denoted as $sm_{j}^{gk}$, which can be calculated using the following the formula:

$$sm_{j}^{gk}=\frac{1}{m-1}\sum_{h=1,h\neq g}^{m}\frac{1}{\frac{1}{n}\sum_{i=1}^{n}d_{ij}^{gh}(t_{k})}$$ (15)

where $d_{ij}^{gk}(t_{{}_{k}})$ represents the distance between $L_{ij}^{gk}(p)$ and $L_{ij}^{hk}(p)$.

**Step 3-1-3.** Calculate the credibility degree of DMs.

The credibility of DMs is determined by both hesitancy and similarity of their evaluations. The lower the hesitancy degree and the higher the similarity degree, the more credible the DMs’ evaluations are considered to be. The credibility degree of *c*_*g*_ is denoted as $cd_{j}^{gk}$, and it can be calculated by combining the hesitancy degree and the similarity degree using the following formula:

$$cd_{j}^{gk}=\sqrt{sm_{j}^{gk}\times (1-hd_{j}^{gk})}$$ (16)

**Step 3-1-4.** Calculate the weights of DMs.

The weights assigned to DMs are determined based on their credibility degree. The weight of *c*_*g*_ for attribute *x*_*j*_ in period *t*_*k*_ is denoted as $\omega_{j}^{gk}$, and the weight of *c*_*g*_ for reward-punishment intentions on attribute *x*_*j*_ is denoted as $\omega_{j}^{g}$. These weights can be calculated using the following formulas:

$$\omega_{j}^{gk}=\frac{cd_{j}^{gk}}{\sum_{g=1}^{e}cd_{j}^{gk}}$$ (17)

$$\omega_{j}^{g}=\frac{\sum_{k=1}^{q}cd_{j}^{gk}}{\sum_{g=1}^{e}\sum_{k=1}^{q}cd_{j}^{gk}}$$ (18)

**Step 3-1-5.** Aggregate the evaluations of all DMs.

Based on the PLWA operator, the group evaluation of *o*_*i*_ for attribute *x*_*j*_ in period *t*_*k*_ can be obtained by aggregating the evaluations of all DMs as follows:

$$\begin{array}{l} L_{ij}^{{}_{k}}(p)=\left\{L_{ij,k}^{\left(l\right)}(p_{ij,k}^{\left(l\right)})|L_{ij,k}^{\left(l\right)}\in S,l=1,2,\cdots ,\#L_{ij,k}\right\}\\ \;=\mathrm{PLWA}(L_{ij}^{1k}(p),L_{ij}^{2k}(p),\cdots ,L_{ij}^{ek}(p))\\ \;=\overset{e}{\underset{g=1}{⊕}}\omega_{j}^{gk}L_{ij}^{gk}(p) \end{array}$$ (19)

Therefore, the probabilistic linguistic group decision matrices of sustainable suppliers are obtained as follows:

$$L_{j}=[\begin{array}{cccc} L_{1j}^{1}(p) & L_{1j}^{2}(p) & \cdots & L_{1j}^{q}(p)\\ L_{2j}^{1}(p) & L_{2j}^{2}(p) & \cdots & L_{2j}^{q}(p)\\ \vdots & \vdots & \ddots & \vdots \\ L_{nj}^{1}(p) & L_{nj}^{2}(p) & \cdots & L_{nj}^{q}(p) \end{array}]$$ (20)

where $L_{ij}^{k}(p)$ is the probabilistic linguistic group evaluation of *o*_*i*_ for attribute *x*_*j*_ in period *t*_*k*_.

Based on the PLWA operator, the group reward-punishment intentions for attribute *x*_*j*_ can be obtained by aggregating the reward-punishment intention evaluations of all DMs as follows:

$$\begin{array}{l} {\tilde{L}}_{j}(\tilde{p})=\left\{{\tilde{L}}_{j}({\tilde{p}}_{j}^{\left(\tilde{l}\right)})|{\tilde{L}}_{j}^{\left(\tilde{l}\right)}\in S,\tilde{l}=1,2,\cdots ,\#{\tilde{L}}_{j}\right\}\\ \;=\mathrm{PLWA}({\tilde{L}}_{1}(\tilde{p}),{\tilde{L}}_{2}(\tilde{p}),\cdots ,{\tilde{L}}_{e}(\tilde{p}))\\ \;=\overset{e}{\underset{g=1}{⊕}}\omega_{j}^{g}{\tilde{L}}_{g}(\tilde{p}) \end{array}$$ (21)

Therefore, the probabilistic linguistic decision matrix of group reward-punishment intentions is obtained as follows:

$$\tilde{L}=[{\tilde{L}}_{1}(\tilde{p}),{\tilde{L}}_{2}(\tilde{p}),\cdots ,{\tilde{L}}_{m}(\tilde{p})]$$ (22)

where ${\tilde{L}}_{j}(\tilde{p})$ is the evaluation of group reward-punishment intentions for attribute *x*_*j*_.

### Aggregate the group evaluations of different periods based on PLTOI operator

In the process of sustainable supplier selection, to select the sustainable supplier that meets the DMs’ expectations of "rapid and stable development", it is necessary to assess the development trend of sustainable suppliers based on DMs’ intentions. The trend change level and the trend change stability are two important aspects to measure the development trend. At the same time, to make a comprehensive and reasonable judgment, it is essential to comprehensively consider the development state and trend of sustainable suppliers to conduct multi-stage information aggregation.

#### Step 4

Calculate the degree of preference for reward or punishment.

The degree of preference for reward or punishment corresponding to the $\tilde{l}\mathrm{th}$ linguistic term ${\tilde{L}}^{\left(\tilde{l}\right)}$ in the evaluation of group reward-punishment intentions is denoted as $\mu_{\tilde{l}}$, and it can be calculated using the following formula:

$$\mu_{\tilde{l}}=\frac{{\tilde{r}}^{\left(\tilde{l}\right)}}{2\tau }+\frac{1}{2}$$ (23)

where ${\tilde{r}}^{\left(\tilde{l}\right)}$ represents the subscript of the linguistic term ${\tilde{L}}^{\left(\tilde{l}\right)}$. According to the Formula (23), it can be observed that the more DMs prefer punishment, the smaller the degree of preference for reward or punishment; the more DMs prefer reward, the greater the degree of preference for reward or punishment.

DMs control the incentive points based on their degree of preference for reward or punishment, which allows them to implement the means of reward incentives or punishment incentives for sustainable suppliers. When DMs prefer reward, setting incentive points enables most sustainable suppliers to receive reward incentives; when DMs prefer punishment, setting incentive points enables most sustainable suppliers to receive punishment incentives; When DMs do not prioritize reward and punishment, they can set incentive points in a manner that only a limited number of sustainable suppliers receive reward incentives or punishment incentives.

**Example 1.** Assume the group reward-punishment intentions for attribute *x*_*j*_ are ${\tilde{L}}_{j}(\tilde{p})=\{{\tilde{s}}_{1.2}(0.7),{\tilde{s}}_{0.6}(0.3)\}$.

According to Formula (23), the degree of preference for reward or punishment can be calculated: $\mu_{1}=\frac{1.2}{2\times 3}+\frac{1}{2}=0.7$, $\mu_{2}=\frac{0}{2\times 3}+\frac{1}{2}=0.5$.

#### Step 5

Measure the trend change level.

The trend change level of sustainable suppliers is mainly reflected in the probability gain level of various linguistic terms in the group evaluations. On this basis, an incentive coefficient reflecting the trend change level of sustainable suppliers is obtained. This coefficient is derived by implementing reward incentives or punishment incentives for sustainable suppliers according to the group reward-punishment intentions.

**Step 5–1.** Calculate the probability gain level.

For the group evaluation of *o*_*i*_ regarding attribute *x*_*j*_ in period *t*_*k*_, the probability gain level of the *l*th linguistic term *L*^(*l*)^ is denoted as $d_{ij,k}^{l}$, and it can be calculated using the following formula:

$$d_{ij,k}^{l}=p_{ij,k}^{\left(l\right)}-p_{ij,k-1}^{\left(l\right)}(k=2,3,\cdots ,q)$$ (24)

For the evaluations of all sustainable suppliers for attribute *x*_*j*_ in period *t*_*k*_, the maximum probability gain level, the minimum probability gain level and the average probability gain level of the *l*th linguistic term *L*^(*l*)^ are respectively denoted as $d_{l,jk}^{\max}$, $d_{l,jk}^{\min}$, $d_{l,jk}^{\mathrm{ave}}$, and they can be calculated using the following formulas:

$$\left\{ \begin{array}{l} d_{l,jk}^{\max}=\underset{i}{\max}(d_{ij,k}^{l})\\ d_{l,jk}^{\min}=\underset{i}{\min}(d_{ij,k}^{l})\\ d_{l,jk}^{\mathrm{ave}}=\frac{1}{n}\sum_{i=1}^{n}d_{ij,k}^{l} \end{array}\right.$$ (25)

**Example 2.** Assume the group evaluations of *o*_1_ and *o*_2_ for attribute *x*_*j*_ in period *t*_1_ are $L_{1j}^{{}_{1}}(p)=\{s_{1.8}(0.8),s_{-0.6}(0.2)\}$, $L_{2j}^{{}_{1}}(p)=\{s_{1.8}(0.7),s_{-0.6}(0.3)\}$, and the group evaluations in period *t*_2_ are $L_{1j}^{2}(p)=\{s_{1.8}(0.6),s_{-0.6}(0.4)\}$, $L_{2j}^{{}_{2}}(p)=\{s_{1.8}(0.8),s_{-0.6}(0.2)\}$.

According to Formula (24), the probability gain level can be calculated:

$d_{1j,2}^{1}=0.6-0.8=-0.2$, $d_{1j,2}^{2}=0.4-0.2=0.2$; $d_{2j,2}^{1}=0.8-0.7=0.1$, $d_{2j,2}^{2}=0.2-0.3=-0.1$.

Then, according to Formula (25), calculate the maximum probability gain level, the minimum probability gain level and the average probability gain level:

$d_{1,j2}^{\max}=\underset{i}{\max}(-0.2,0.1)=0.1$, $d_{1,j2}^{\min}=\underset{i}{\min}(-0.2,0.1)=-0.2$, $d_{1,j2}^{ave}=\frac{1}{2}(-0.2+0.1)=-0.05$;

$d_{2,j2}^{\max}=\underset{i}{\max}(0.2,-0.1)=0.2$, $d_{2,j2}^{\min}=\underset{i}{\min}(0.2,-0.1)=-0.1$, $d_{2,j2}^{ave}=\frac{1}{2}(0.2+(-0.1))=0.05$ **Step 5–2.** Calculate the positive incentive points and the negative incentive points of probability gain level.

When the group reward-punishment intention falls into the $\tilde{l}\mathrm{th}$ linguistic term ${\tilde{L}}^{\left(\tilde{l}\right)}$, for the group evaluations of sustainable suppliers regarding attribute *x*_*j*_ in period *t*_*k*_, if the subscript *r*^(*l*)^ of the *l*th linguistic term *L*^(*l*)^ satisfies *r*^(*l*)^≥0, the positive incentive point and the negative incentive point of probability gain level are respectively denoted as $d_{l,jk}^{\tilde{l}+}$, $d_{l,jk}^{\tilde{l}-}$, and they can be calculated using the following formulas:

$$\left\{ \begin{array}{l} d_{l,jk}^{\tilde{l}+}=d_{l,jk}^{\mathrm{ave}}+(d_{l,jk}^{\max}-d_{l,jk}^{\mathrm{ave}})(1-\mu_{\tilde{l}})\\ d_{l,jk}^{\tilde{l}-}=d_{l,jk}^{\mathrm{ave}}-(d_{l,jk}^{\mathrm{ave}}-d_{l,jk}^{\min})\mu_{\tilde{l}} \end{array}\right.$$ (26)

If the subscript *r*^(*l*)^ of the *l*th linguistic term *L*^(*l*)^ satisfies *r*^(*l*)^<0, the positive incentive point and the negative incentive point of probability gain level are respectively denoted as ${\hat{d}}_{l,jk}^{\tilde{l}+}$, ${\hat{d}}_{l,jk}^{\tilde{l}-}$, and they can be calculated using the following formulas:

$$\left\{ \begin{array}{l} {\hat{d}}_{l,jk}^{\tilde{l}+}=d_{l,jk}^{\mathrm{ave}}-(d_{l,jk}^{\mathrm{ave}}-d_{l,jk}^{\min})(1-\mu_{\tilde{l}})\\ {\hat{d}}_{l,jk}^{\tilde{l}-}=d_{l,jk}^{\mathrm{ave}}+(d_{l,jk}^{\max}-d_{l,jk}^{\mathrm{ave}})\mu_{\tilde{l}} \end{array}\right.$$ (27)

According to Formulas (26)–(27), when the subscript *r*^(*l*)^ satisfies *r*^(*l*)^≥0, the positive incentive point of probability gain level is greater than the negative incentive point of probability gain level; when the subscript *r*^(*l*)^ satisfies *r*^(*l*)^<0, the positive incentive point of probability gain level is less than the negative incentive point of probability gain level.

**Example 3.** In the context of Example 1 and Example 2, assume the group reward-punishment intention falls into the first linguistic term ${\tilde{s}}_{1.2}$.

According to Formulas (26)–(27), we can get the incentive points of probability gain level for the linguistic terms *s*_1.8_ and *s*_−0.6_:

$$d_{1,j2}^{1+}=-0.05+(0.1-(-0.05))\times (1-0.7)=-0.0050,$$

$$d_{1,j2}^{1-}=-0.05-(-0.05-(-0.2))\times 0.7=-0.1550,$$

$${\hat{d}}_{2,j2}^{1+}=0.05-(0.05-(-0.1))\times (1-0.7)=0.0050,$$

$${\hat{d}}_{2,j2}^{1-}=0.05+(0.2-0.05)\times 0.7=0.1550.$$

**Step 5–3.** Calculate the total incentives of probability gain level.

After introducing the incentive points of the probability gain level, for the linguistic term *L*^(*l*)^ that its subscript *r*^(*l*)^ satisfies *r*^(*l*)^≥0, the positive incentives and the negative incentives of probability gain level obtained by *o*_*i*_ for attribute *x*_*j*_ in period *t*_*k*_ are respectively denoted as $u_{ij,l}^{\tilde{l}+}(t_{k})$, $u_{ij,l}^{\tilde{l}-}(t_{k})$, and they can be calculated using the following formulas:

$$u_{ij,l}^{\tilde{l}+}(t_{k})=\{\begin{array}{ll} (d_{ij,k}^{l}-d_{l,jk}^{\tilde{l}+})r^{\left(l\right)} & d_{ij,k}^{l}>d_{l,jk}^{\tilde{l}+}\\ 0 & others \end{array}$$ (28)

$$u_{ij,l}^{\tilde{l}-}(t_{k})=\{\begin{array}{ll} (d_{ij,k}^{l}-d_{l,jk}^{\tilde{l}-})r^{\left(l\right)} & d_{ij,k}^{l}<d_{l,jk}^{\tilde{l}-}\\ 0 & others \end{array}$$ (29)

For the linguistic term *L*^(*l*)^ that its subscript *r*^(*l*)^ satisfies *r*^(*l*)^<0, the positive incentives and the negative incentives of probability gain level obtained by *o*_*i*_ for attribute *x*_*j*_ in period *t*_*k*_ are respectively denoted as ${\hat{u}}_{ij,l}^{\tilde{l}+}(t_{k})$, ${\hat{u}}_{ij,l}^{\tilde{l}-}(t_{k})$, and they can be calculated using the following formulas:

$${\hat{u}}_{ij,l}^{\tilde{l}+}(t_{k})=\{\begin{array}{ll} (d_{ij,k}^{l}-{\hat{d}}_{l,jk}^{\tilde{l}+})r^{\left(l\right)} & d_{ij,k}^{l}<{\hat{d}}_{l,jk}^{\tilde{l}+}\\ 0 & others \end{array}$$ (30)

$${\hat{u}}_{ij,l}^{\tilde{l}-}(t_{k})=\{\begin{array}{ll} (d_{ij,k}^{l}-{\hat{d}}_{l,jk}^{\tilde{l}-})r^{\left(l\right)} & d_{ij,k}^{l}>{\hat{d}}_{l,jk}^{\tilde{l}-}\\ 0 & others \end{array}$$ (31)

When the group reward-punishment intention falls into the $\tilde{l}\mathrm{th}$ linguistic term ${\tilde{L}}^{\left(\tilde{l}\right)}$, the total incentives of probability gain level obtained by *o*_*i*_ for attribute *x*_*j*_ in period *t*_*k*_ is denoted as $u_{ij,k}^{\tilde{l}}$, and it can be calculated using the following formula:

$$u_{ij,k}^{\tilde{l}}=\sum_{l=1}^{\#L}\left(u_{ij,l}^{\tilde{l}+}(t_{k})+u_{ij,l}^{\tilde{l}-}(t_{k})+{\hat{u}}_{ij,l}^{\tilde{l}+}(t_{k})+{\hat{u}}_{ij,l}^{\tilde{l}-}(t_{k})\right)$$ (32)

**Example 4.** In the context of Example 1 and Example 3.

According to Formulas (28)–(32), the total incentives of probability gain level for *o*_1_ and *o*_2_ can be calculated as follows:

$$u_{1j,\;1}^{1+}(t_{2})=0,u_{1j,1}^{1-}(t_{2})=(-0.2-(-0.1550))\times 1.8=-0.0810,$$

$${\hat{u}}_{1j,2}^{1+}(t_{2})=0,\;{\hat{u}}_{1j,2}^{1-}(t_{2})=(0.2-0.1550)\times (-0.6)=-0.0270,$$

$$u_{1j,2}^{1}=0+(-0.0810)+0+(-0.0270)=-0.1080;$$

$$u_{2j,1}^{1+}(t_{2})=(0.1-(-0.0050))\times 1.8=0.1890,\;u_{2j,1}^{1-}(t_{2})=0,$$

$${\hat{u}}_{2j,2}^{1+}(t_{2})=(-0.1-0.0050)\times (-0.6)=0.0630,\;{\hat{u}}_{2j,2}^{1-}(t_{2})=0,$$

$$u_{2j,2}^{1}=0.1890+0+0.0630+0=0.2520.$$

**Fig 2:**
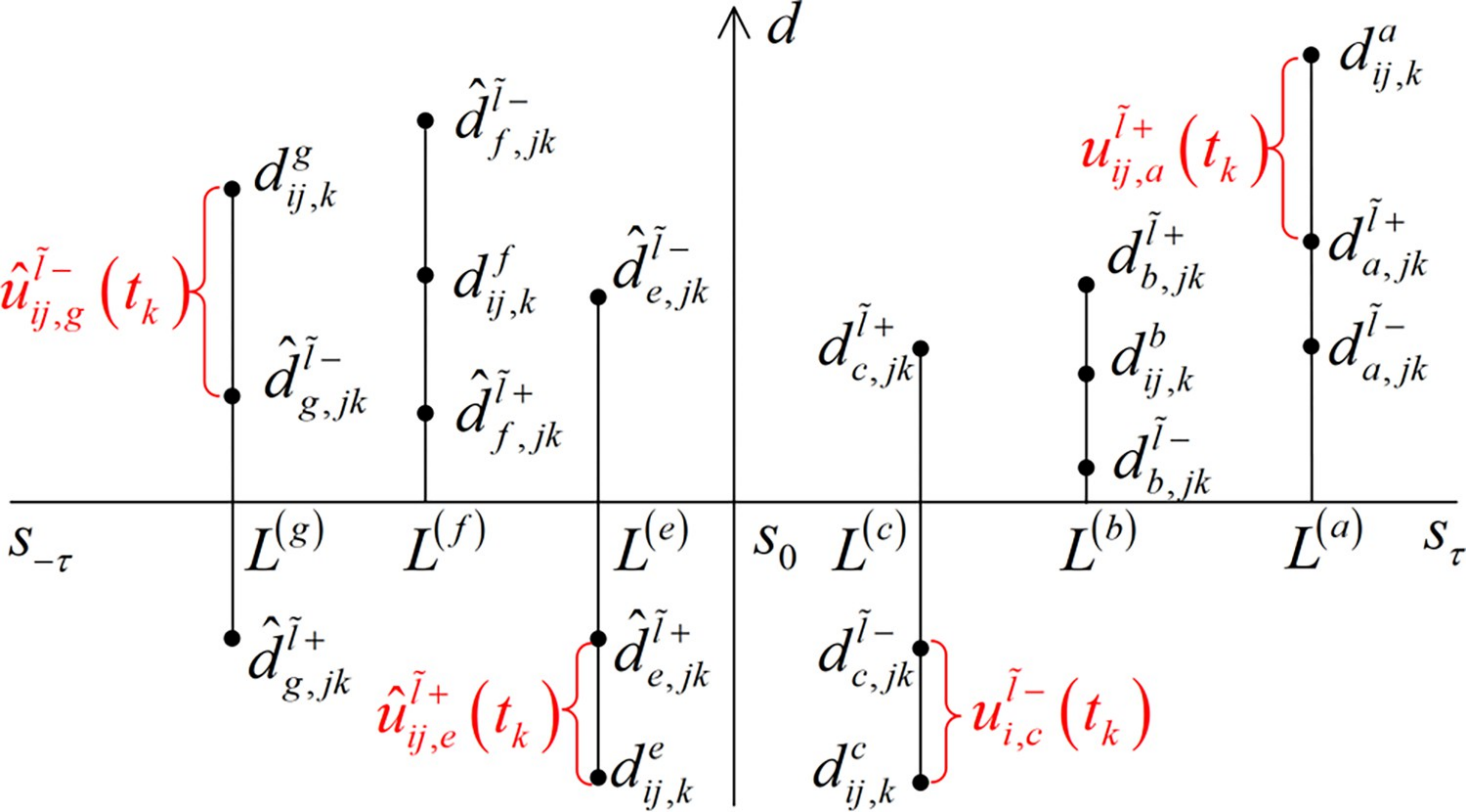
Geometric graphics of incentives for probability gain level.

The positive incentives and the negative incentives of probability gain level are depicted geometrically in Fig 2. This figure shows the potential positioning of probability gain level ($d_{ij,k}^{l}$) to the positive incentive points ($d_{l,jk}^{\tilde{l}+}$ or ${\hat{d}}_{l,jk}^{\tilde{l}+}$) and the negative incentive points ($d_{l,jk}^{\tilde{l}-}$ or ${\hat{d}}_{l,jk}^{\tilde{l}-}$). *L*^(*a*)^(*r*^(*a*)^≥0) and *L*^(*e*)^(*r*^(*e*)^<0), *L*^(*b*)^(*r*^(*b*)^≥0) and *L*^(*f*)^(*r*^(*f*)^<0), *L*^(*c*)^(*r*^(*c*)^≥0) and *L*^(*g*)^(*r*^(*g*)^<0) respectively denote the linguistic terms which sustainable supplier (*o*_*i*_) receive positive, zero and negative incentives of probability gain level, and these incentives are denoted by $u_{ij,a}^{\tilde{l}+}(t_{k})$, ${\hat{u}}_{ij,e}^{\tilde{l}+}(t_{k})$, 0, 0, $u_{ij,c}^{\tilde{l}-}(t_{k})$ and ${\hat{u}}_{ij,g}^{\tilde{l}-}(t_{k})$ respectively.

**Step 5–4.** Calculate the incentive coefficient of trend change level.

When the group reward-punishment intention falls into the $\tilde{l}\mathrm{th}$ linguistic term ${\tilde{L}}^{\left(\tilde{l}\right)}$, the incentive coefficient of trend change level of *o*_*i*_ for attribute *x*_*j*_ in period *t*_*k*_ is denoted as $\rho_{ij,k}^{\tilde{l}}$, and it can be calculated using the following formula:

$$\rho_{ij,k}^{\tilde{l}}=\{\begin{array}{ll} \frac{2}{1+\exp(-u_{ij,k}^{\tilde{l}})} & u_{ij,k}^{\tilde{l}}>0\\ 1 & u_{ij,k}^{\tilde{l}}=0\\ \frac{2}{1+\exp(-u_{ij,k}^{\tilde{l}})} & u_{ij,k}^{\tilde{l}}<0 \end{array}$$ (33)

According to Formula (33), $\rho_{ij,k}^{\tilde{l}}$ is a monotonically increasing function, and $\rho_{ij,k}^{\tilde{l}}$ increases as $u_{ij,k}^{\tilde{l}}$ increases. The function graph of $\rho_{ij,k}^{\tilde{l}}$ has an inflection point. Before the inflection point, $\rho_{ij,k}^{\tilde{l}}$ grows faster and faster, while after the inflection point, $\rho_{ij,k}^{\tilde{l}}$ grows slower and slower. This inherent feature of the function can reflect the guiding idea of "moderate growth" for sustainable suppliers.

**Example 5.** In the context of Example 4.

According to Formula (33), we can get the incentive coefficients of trend change level for *o*_1_ and *o*_2_:

$$\rho_{1j,2}^{1}=\frac{2}{1+\exp(-(-0.1080))}=0.9461,\;\rho_{2j,2}^{1}=\frac{2}{1+\exp(-0.2520)}=1.1253.$$

#### Step 6

Measure the trend change stability.

**Step 6–1.** Calculate probability gain stability.

For the group evaluations of *o*_*i*_ for attribute *x*_*j*_ in all periods, the probability gain stability of the *l*th linguistic term *L*^(*l*)^ is denoted as *SD*_*ij*,*l*_, and it can be calculated using the following formula:

$$SD_{ij,l}=\frac{1}{{\left(\sum_{k=2}^{T}{\left(d_{ij,k}^{l}-{\bar{d}}_{ij}^{l}\right)}^{2}/(T-2)\right)}^{\frac{1}{2}}+1}$$ (34)

where ${\bar{d}}_{ij}^{l}$ represents the average probability gain of the *l*th linguistic term *L*^(*l*)^ for the group evaluation of *o*_*i*_ for attribute *x*_*j*_ in all periods.

For the evaluations of all sustainable suppliers for attribute *x*_*j*_, the maximum probability gain stability, the minimum probability gain stability and the average probability gain stability of the *l*th linguistic term *L*^(*l*)^ are respectively denoted as $SD_{l,j}^{\max}$, $SD_{l,j}^{\min}$, $SD_{l,j}^{\mathrm{ave}}$, and they can be calculated using the following formulas:

$$\left\{ \begin{array}{l} SD_{l,j}^{\max}=\underset{i}{\max}(SD_{ij,l})\\ SD_{l,j}^{\min}=\underset{i}{\min}(SD_{ij,l})\\ SD_{l,j}^{\mathrm{ave}}=\frac{1}{n}\sum_{i=1}^{n}SD_{ij,l} \end{array}\right.$$ (35)

**Example 6.** In the context of Example 2, assume the group evaluations of *o*_1_ and *o*_2_ for attribute *x*_*j*_ in period *t*_3_ are $L_{1j}^{3}(p)=\{s_{1.8}(0.5),s_{-0.6}(0.5)\}$, $L_{2j}^{{}_{3}}(p)=\{s_{1.8}(0.6),s_{-0.6}(0.4)\}$.

According to Formula (34), the probability gain stability can be calculated as follows:

$$SD_{1j,1}=\frac{1}{{\left(\left({\left((-0.2)-\frac{\left(-0.2)+(-0.1\right)}{2}\right)}^{2}+{\left((-0.1)-\frac{\left(-0.2)+(-0.1\right)}{2}\right)}^{2})/(3-2\right)\right)}^{\frac{1}{2}}+1}=0.9340,$$

$$SD_{1j,2}=\frac{1}{{\left(\left({\left(0.2-\frac{0.2+0.1}{2}\right)}^{2}+{\left(0.1-\frac{0.2+0.1}{2}\right)}^{2})/(3-2\right)\right)}^{\frac{1}{2}}+1}=0.9340,$$

$$SD_{2j,1}=\frac{1}{{\left(\left({\left(0.1-\frac{0.1+(-0.2)}{2}\right)}^{2}+{\left((-0.2)-\frac{0.1+(-0.2)}{2}\right)}^{2})/(3-2\right)\right)}^{\frac{1}{2}}+1}=0.8250,$$

$$SD_{2j,1}=\frac{1}{{\left(\left({\left((-0.1)-\frac{(-0.1)+0.2}{2}\right)}^{2}+{\left(0.2-\frac{(-0.1)+0.2}{2}\right)}^{2})/(3-2\right)\right)}^{\frac{1}{2}}+1}=0.8250.$$

Then, according to Formula (35), calculate the maximum probability gain stability, the minimum probability gain stability and the average probability gain stability:

$$SD_{1,j}^{\max}=\underset{i}{\max}(0.9340,\;0.8250)=0.9340,SD_{1,j}^{\min}=\underset{i}{\min}(0.9340,0.8250)=0.8250,$$

$$SD_{1,j}^{ave}=\frac{0.9340+0.8250}{2}=0.8795;\;SD_{2,j}^{\max}=\underset{i}{\max}(0.9340,0.8250)=0.9340,$$

$$SD_{2,j}^{\min}=\underset{i}{\min}(0.9340,0.8250)=0.8250,\;SD_{2,j}^{ave}=\frac{0.9340+0.8250}{2}=0.8795.$$

**Step 6–2.** Calculate the positive incentive points and the negative incentive points of probability gain stability.

When the group reward-punishment intention falls into the $\tilde{l}\mathrm{th}$ linguistic term ${\tilde{L}}^{\left(\tilde{l}\right)}$, for the *l*th linguistic term *L*^(*l*)^ in the group evaluations of sustainable suppliers regarding attribute *x*_*j*_, the positive incentive point and the negative incentive point of probability gain stability are respectively denoted as $\varphi_{l,j}^{\tilde{l}+}$, $\varphi_{l,j}^{\tilde{l}+}$, and they can be calculated using the following formulas:

$$\left\{ \begin{array}{l} \varphi_{l,j}^{\tilde{l}+}=SD_{l,j}^{\mathrm{ave}}+(SD_{l,j}^{\max}-SD_{l,j}^{\mathrm{ave}})(1-\mu_{\tilde{l}})\\ \varphi_{l,j}^{\tilde{l}-}=SD_{l,j}^{\mathrm{ave}}-(SD_{l,j}^{\mathrm{ave}}-SD_{l,j}^{\min})\mu_{\tilde{l}} \end{array}\right.$$ (36)

**Example 7.** In the context of Example 1 and Example 6, assume the group reward-punishment intention falls into the first linguistic term ${\tilde{s}}_{1.2}$.

According to Formula (36), we can calculate the incentive points of probability gain stability for linguistic terms *s*_1.8_ and *s*_−0.6_:

$$\varphi_{1,j}^{1+}=0.8795+(0.9340-0.8795)\times (1-0.7)=0.8958,$$

$$\varphi_{1,j}^{1-}=0.8795-(0.8795-0.8250)\times 0.7=0.8413;$$

$$\varphi_{2,j}^{1+}=0.8795+(0.9340-0.8795)\times (1-0.7)=0.8958,$$

$$\varphi_{2,j}^{1-}=0.8795-(0.8795-0.8250)\times 0.7=0.8413.$$

**Step 6–3.** Calculate the total incentives of probability gain stability.

After introducing the incentive point of the probability gain stability, for the $\tilde{l}\mathrm{th}$ linguistic term ${\tilde{L}}^{\left(\tilde{l}\right)}$, the positive incentives and the negative incentives of probability gain stability obtained by *o*_*i*_ for attribute *x*_*j*_ are denoted as $v_{ij,l}^{\tilde{l}+}$ and $v_{ij,l}^{\tilde{l}-}$, and they can be calculated using the following formulas:

$$v_{ij,l}^{\tilde{l}+}=\{\begin{array}{ll} SD_{ij,l}-\varphi_{l,j}^{\tilde{l}+} & SD_{ij,l}>\varphi_{l,j}^{\tilde{l}+}\\ 0 & others \end{array}$$ (37)

$$v_{ij,l}^{\tilde{l}-}=\{\begin{array}{ll} SD_{ij,l}-\varphi_{l,j}^{\tilde{l}-} & SD_{ij,l}<\varphi_{l,j}^{\tilde{l}-}\\ 0 & others \end{array}$$ (38)

When the group reward-punishment intention falls into the $\tilde{l}\mathrm{th}$ linguistic term ${\tilde{L}}^{\left(\tilde{l}\right)}$, the total incentives of probability gain stability obtained by *o*_*i*_ for attribute *x*_*j*_ is denoted as $v_{ij}^{\tilde{l}}$, and it can be calculated using the following formula:

$$v_{ij}^{\tilde{l}}=\sum_{l=1}^{\#L}\left(v_{ij,l}^{\tilde{l}+}+v_{ij,l}^{\tilde{l}-}\right)$$ (39)

**Example 8.** In the context of Example 6 and Example 7.

According to Formulas (37)–(39), the total incentives of probability gain stability for *o*_1_ and *o*_2_ can be calculated:

$$v_{1j,1}^{1+}=0.9340-0.8958=0.0381,\;v_{1j,1}^{1-}=0,$$

$$v_{1j,2}^{1+}=0.9340-0.8958=0.0381,\;v_{1j,2}^{1-}=0,$$

$$v_{1j}^{1}=0.0381+0+0.0381+0=0.0762;$$

$$v_{2j,1}^{1+}=0,\;v_{2j,1}^{1-}=0.8250-0.8413=-0.0163,$$

$$v_{2j,2}^{1+}=0,v_{2j,2}^{1-}=0.8250-0.8413=-0.0163,$$

$$v_{2j}^{1}=-0.0163+0+(-0.0163)+0=-0.0326.$$

**Fig 3:**
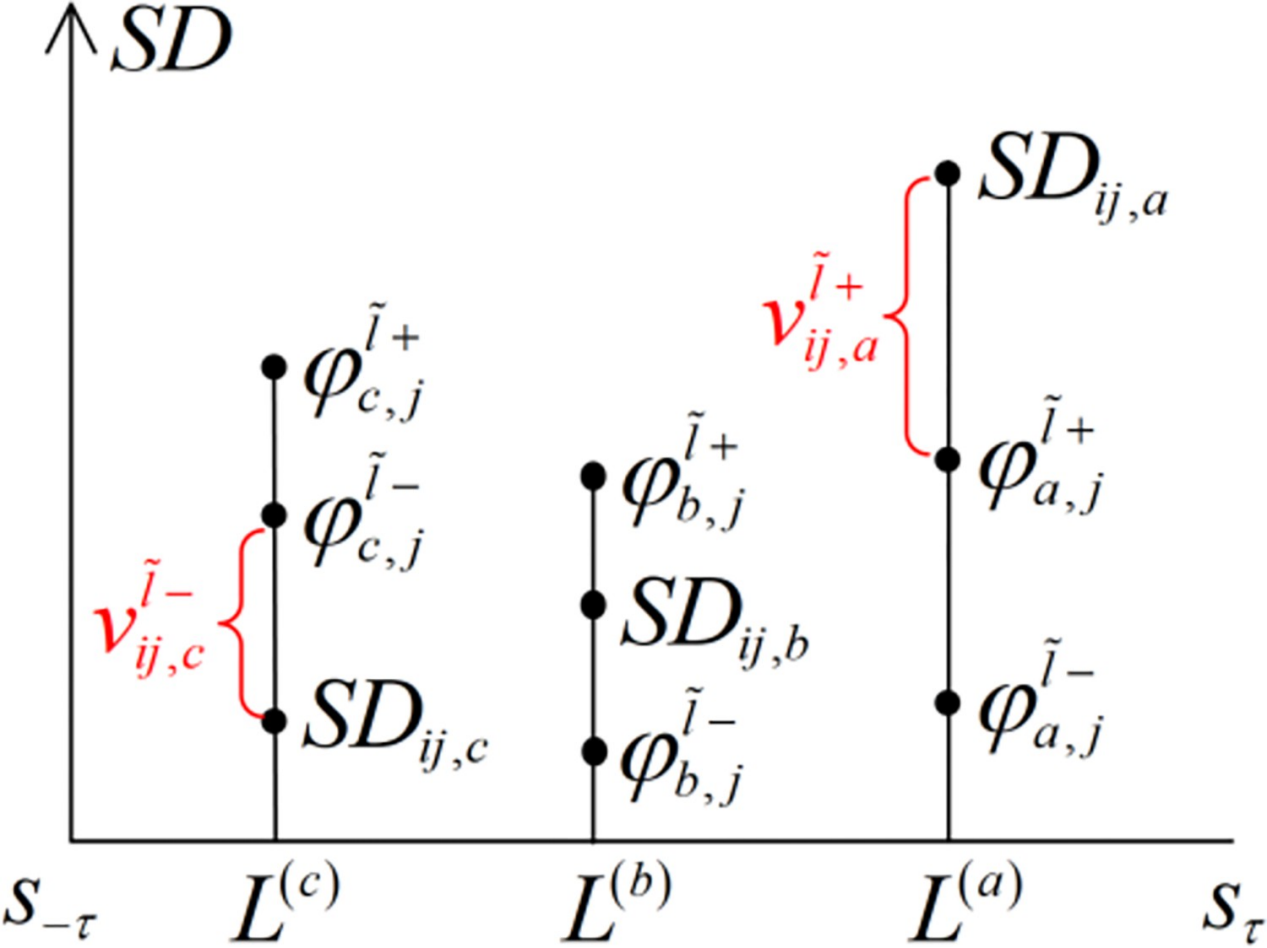
Geometric graphics of incentives for probability gain stability.

The positive incentives and the negative incentives of probability gain stability are shown geometrically in Fig 3. This figure shows the potential positioning of probability gain stability (*SD*_*ij*,*l*_) to the positive incentive points ($\varphi_{l,j}^{\tilde{l}+}$) and the negative incentive points ($\varphi_{l,j}^{\tilde{l}+}$). *L*^(*a*)^, *L*^(*b*)^ and *L*^(*c*)^ respectively denote the linguistic terms which sustainable suppliers (*o*_*i*_) receive positive, zero and negative incentives of probability gain stability, and these incentives are denoted by $v_{ij,a}^{\tilde{l}+}$, 0 and $v_{ij,c}^{\tilde{l}-}$ respectively.

**Step 6–4.** Calculate the incentive coefficient of trend change stability.

When the group reward-punishment intention falls into the $\tilde{l}\mathrm{th}$ linguistic term ${\tilde{L}}^{\left(\tilde{l}\right)}$, the incentive coefficient of trend change stability of *o*_*i*_ for attribute *x*_*j*_ is denoted as $\theta_{ij}^{\tilde{l}}$, and it can be calculated using the following formula:

$$\theta_{ij}^{\tilde{l}}=\{\begin{array}{ll} \frac{2}{1+\exp(-v_{ij}^{\tilde{l}})} & v_{ij}^{\tilde{l}}>0\\ 1 & v_{ij}^{\tilde{l}}=0\\ \frac{2}{1+\exp(-v_{ij}^{\tilde{l}})} & v_{ij}^{\tilde{l}}<0 \end{array}$$ (40)

According to Formula (40), $\theta_{ij}^{\tilde{l}}$ is a monotonically increasing function, and $\theta_{ij}^{\tilde{l}}$ increases as $v_{ij}^{\tilde{l}}$ increases. The essence of function $\theta_{ij}^{\tilde{l}}$ is to provide incentives to sustainable suppliers according to trend change stability in all periods, which can reflect the guiding idea of "stable development" for sustainable suppliers.

**Example 9.** In the context of Example 8.

According to Formula (40), we can calculate the incentive coefficients of trend change stability for *o*_1_ and *o*_2_:

$$\theta_{1j}^{1}=\frac{2}{1+\exp(-0.0762)}=1.0381,\;\theta_{2j}^{1}=\frac{2}{1+\exp(-(-0.0326))}=0.9837.$$

#### Step 7

Aggregate the probabilistic linguistic group evaluations of different periods.

**Step 7–1.** Calculate the composite incentive coefficient of trend change.

When the group reward-punishment intention falls into the $\tilde{l}\mathrm{th}$ linguistic term ${\tilde{L}}^{\left(\tilde{l}\right)}$, the composite incentive coefficient of trend change of *o*_*i*_ for attribute *x*_*j*_ in period *t*_*k*_ is denoted by $\delta_{ij,k}^{\tilde{l}}$, and it can be calculated by combining the incentive coefficient of trend change level and the incentive coefficient of trend change stability. The formula is as follows:

$$\delta_{ij,k}^{\tilde{l}}=\{\begin{array}{ll} 1 & k=1\\ \rho_{ij,k}^{\tilde{l}}\theta_{ij}^{\tilde{l}} & k=2,3,\cdots ,q \end{array}$$ (41)

**Step 7–2.** Aggregate the group evaluations of all periods.

To comprehensively consider the development state and trend of sustainable suppliers in each period, and aggregate the evaluations of all periods, a new aggregation operator is proposed.

**Definition 8.** Let $L_{i}^{t_{1}}(p),L_{i}^{t_{2}}(p),\cdots ,L_{i}^{t_{q}}(p)$ be a set of time-ordered PLTS that need to be aggregated, *ε*_*k*_ be the time-inducible component, $\varsigma^{\left(\tilde{l}\right)}$ be the reward-punishment preference-inducible component, and $\xi_{i,k}^{\tilde{l}}$ be the incentive-inducible component. Then an aggregation operator named probabilistic linguistic time-ordered incentive (PLTOI) is shown as follows:

$$\mathrm{PLTOI}\{L_{i}^{t_{1}}(p),L_{i}^{t_{2}}(p),\cdots ,L_{i}^{t_{T}}(p)\}=\overset{T}{\underset{k=1}{⊕}}\varepsilon_{k}(\overset{\#\tilde{L}}{\underset{\tilde{l}=1}{⊕}}\varsigma^{\left(\tilde{l}\right)}(\xi_{i,k}^{\tilde{l}}L_{i}^{t_{k}}(p)))$$ (42)

where *ε* = (*ε*_1_,*ε*_2_,⋯,*ε*_*q*_)^*T*^ is the time weight vector, and it is usually determined by the time function that lays more stress on the present evaluations than on the past [63]; in addition, if there is no specific time preference, let *ε*_*k*_ (*k* = 1,2,⋯,*q*) be equal to $\frac{1}{q}$.

Based on the PLTOI operator, the dynamic evaluation of *o*_*i*_ on attribute *x*_*j*_ can be obtained by aggregating the group evaluations of all periods as follows:

$$\begin{array}{l} L_{ij}(p)=\mathrm{PLTOI}\{L_{ij}^{1}(p),L_{ij}^{2}(p),\cdots ,L_{ij}^{q}(p)\}\\ \;=\overset{q}{\underset{k=1}{⊕}}\varepsilon_{k}(\overset{\#\tilde{L}}{\underset{\tilde{l}=1}{⊕}}{\tilde{p}}_{j}^{\left(\tilde{l}\right)}(\delta_{ij,k}^{\tilde{l}}L_{ij}^{k}(p))) \end{array}$$ (43)

Thus, the dynamic group decision matrix of sustainable suppliers is obtained as follows:

$$L=[\begin{array}{cccc} L_{11}(p) & L_{12}(p) & \cdots & L_{1m}(p)\\ L_{21}(p) & L_{22}(p) & \cdots & L_{2m}(p)\\ \vdots & \vdots & \ddots & \vdots \\ L_{n1}(p) & L_{n2}(p) & \cdots & L_{nm}(p) \end{array}]$$ (44)

**Example 10.** Assume the group evaluations of *o*_1_ for attribute *x*_*j*_ in periods *t*_1_, *t*_2_ and *t*_3_ are $L_{1j}^{{}_{1}}(p)=\{s_{1.8}(0.8),s_{-0.6}(0.2)\}$, $L_{1j}^{{}_{2}}(p)=\{s_{1.8}(0.6),s_{-0.6}(0.4)\}$, $L_{1j}^{3}(p)=\{s_{1.8}(0.5),s_{-0.6}(0.5)\}$, the time weight vector is *ε* = (0.2,0.3,0.5)^*T*^, the group reward-punishment intentions are ${\tilde{L}}_{j}(\tilde{p})=\{{\tilde{s}}_{1.2}(0.7),{\tilde{s}}_{0.6}(0.3)\}$, the composite incentive coefficients of trend change corresponding to ${\tilde{s}}_{1.2}$ and ${\tilde{s}}_{0.6}$ in period *t*_1_ are respectively 1 and 1, the composite incentive coefficients of trend change corresponding to ${\tilde{s}}_{1.2}$ and ${\tilde{s}}_{0.6}$ in period *t*_2_ are respectively 0.9821 and 0.9350, and the composite incentive coefficients of trend change corresponding to ${\tilde{s}}_{1.2}$ and ${\tilde{s}}_{0.6}$ in period *t*_2_ are respectively 1.0817 and 1.0580.

According to Formula (43), we can calculate the dynamic evaluation of *o*_1_ on attribute *x*_*j*_:

$$\begin{array}{l} L_{1j}(p)=\mathrm{PLTOI}\{L_{1j}^{1}(p),L_{1j}^{2}(p),L_{1j}^{3}(p)\}\\ \;=0.2\times (0.7\times (1\times L_{1j}^{1}(p))+0.3\times (1\times L_{1j}^{1}(p)))+0.3\times (0.7\times (0.9821\times L_{1j}^{2}(p)))+\\ \;0.3\times (0.9350\times L_{1j}^{2}(p)))+0.5\times (0.7\times (1.0817\times L_{1j}^{3}(p))+0.3\times (1.0580\times L_{1j}^{3}(p)))\\ \;=\{s_{1.8}(0.19),s_{1.2}(0.47),s_{0.6}(0.20),s_{0}(0.13),s_{-0.6}(0.01)\} \end{array}$$

#### Step 8

Sustainable supplier selection process based on the extended TOPSIS method in the probabilistic linguistic environment.

Based on the probabilistic linguistic dynamic group decision matrix *L* = [*L*_*ij*_(*p*)]_*n*×*m*_, we can obtain a sustainable supplier selection result using the extended TOPSIS method.

## Illustrative example

This section aims to assess the efficiency and applicability of the proposed method using an example of sustainable supplier selection for a civil UAV manufacturing enterprise in China.

### The preparation process

In recent years, with the continuous advances in control and automation technology, the unmanned aerial vehicles (UAV) industry has experienced rapid growth. UAVs are high-tech products with wide-ranging applications in fields such as national defense and security, environmental monitoring, and precision agriculture. With the widespread use of UAVs, UAV manufacturing enterprises are constantly emerging. The flight of UAVs cannot be separated from batteries. As the "heart" of UAVs, the quality of batteries directly influences their performance, making it crucial for UAV manufacturing enterprises to carefully select suitable battery suppliers. However, supplier selection is a complex process that involves procurement, logistics, storage, waste disposal, and other procedures, with multiple factors at play. As a result, it can be challenging to identify suppliers that meet all the necessary attribute requirements. Employing more advanced, scientific methods such as Multiple Attribute Group Decision Making (MAGDM) can ensure better supplier selection and promote sustainable development across the UAV manufacturing industry. As a research background, main focus of this study is to apply the proposed method to rank and select the optimal battery suppliers.

In this case study, five enterprises involved in the manufacturing of UAVs’ batteries have the potential to become the sustainable suppliers through the selection process. Set *O* = {*o*_1_,*o*_2_,*o*_3_,*o*_4_,*o*_5_} is used to refer to the five potential sustainable suppliers. Set *C* = {*c*_1_,*c*_2_,*c*_3_} is used to represent three DMs from the enterprise, namely an engineer, a sales manager and an experienced front-line employee. Let *T* = {*t*_1_,*t*_2_,*t*_3_,*t*_4_} be a set of periods, respectively representing the four quarters of a year. According to previous researches [64, 65] and the specific requirements of the enterprise, four main attributes have been identified for evaluation. These attributes include product quality (*x*_1_), product price (*x*_2_), pollutant discharge (*x*_3_), and work safety and health (*x*_4_). It should be noted that *x*_2_, *x*_3_ are cost attributes and *x*_1_, *x*_4_ are revenue attributes.

The linguistic term set for sustainable supplier performance is denoted as *S* = {*s*_−3_ = extremely poor, *s*_−2_ = very poor, *s*_−1_ = poor, *s*_0_ = medium, *s*_1_ = good, *s*_2_ = very good, *s*_3_ = extremely good}. The linguistic term set for DMs’ reward-punishment intentions is denoted as $\tilde{S}=\{{\tilde{s}}_{-3}=\mathrm{extremely}\;\mathrm{preference}\;\mathrm{for}\;\mathrm{punishment},\;{\tilde{s}}_{-2}=\mathrm{strong}$ $\mathrm{preference}\;\mathrm{for}\;\mathrm{punishment},\;{\tilde{s}}_{-1}=\mathrm{preference}\;\mathrm{fo}\;\mathrm{punishment},\;{\tilde{s}}_{0}=\mathrm{preference}\;\mathrm{for}\;\mathrm{neither}$ $\mathrm{punishment}\;\mathrm{nor}\;\mathrm{reward},\;{\tilde{s}}_{1}=\mathrm{preference}\;\mathrm{for}\;\mathrm{reward},\;{\tilde{s}}_{2}=\mathrm{strong}\;\mathrm{preference}\;\mathrm{for}\;\mathrm{reward},$ ${\tilde{s}}_{3}=\mathrm{extremly}\;\mathrm{preference}\;\mathrm{for}\;\mathrm{reward}\}$.

### The decision making process

#### Step 1

Obtain and standardize the decision matrices.

All the DMs give their evaluations of alternatives by the form ${\bar{L}}_{ij}^{{}_{gk}}(p)=\{{\bar{L}}_{ij,gk}^{\left(l\right)}({\bar{p}}_{ij,gk}^{\left(l\right)})|{\bar{L}}_{ij,gk}^{\left(l\right)}\in S,\bar{l}=1,2,\cdots ,\#{\bar{L}}_{ij,gk}\}$. By Formula (13), the original matrices ${\bar{L}}_{gk}$ are standardized into decision matrices $L_{gk}={\left[L_{ij}^{{}_{gk}}(p)\right]}_{5\times 4}(i=1,2,3,4,5j=1,2,3,4)$. Due to space limitation, only the standardized probabilistic linguistic evaluation matrices of alternatives by *c*_1_ $L_{1k}={\left[L_{ij}^{{}_{1k}}(p)\right]}_{5\times 4}(k=1,2,3,4)$ are listed in S1 Table. All the DMs give their evaluations of reward-punishment intentions by the form ${\tilde{L}}_{g}(\tilde{p})=\{{\tilde{L}}_{g}^{\left(\tilde{l}\right)}({\tilde{p}}_{g}^{\left(\tilde{l}\right)})|{\tilde{L}}_{g}^{\left(\tilde{l}\right)}\in \tilde{S},\tilde{l}=1,2,\cdots ,\#{\tilde{L}}_{g}\}$, and the probabilistic linguistic evaluation matrix of reward-punishment intentions by all DMs $\tilde{L}={\left[{\tilde{L}}_{g}(\tilde{p})\right]}_{1\times m}$ are represented in S2 Table.

#### Step 2

Aggregate the probabilistic linguistic evaluations of different DMs.

Based on Formulas (14)–(16) and Formulas (17)–(18), we can calculate the credibility degree of DMs and the weights of DMs respectively. By employing Formula (19), the standardized probabilistic linguistic evaluations of alternatives by three DMs are aggregated into new group decision matrices $L_{j}={\left[L_{ij}^{k}(p)\right]}_{5\times 4}(j=1,2,3,4)$ related to four periods. Due to space limitation, only the probabilistic linguistic group evaluation matrix for attribute *x*_1_ $L_{1}={\left[L_{i1}^{k}(p)\right]}_{5\times 4}$ is listed in S3 Table. By employing Formula (21), the probabilistic linguistic evaluations of DMs’ own reward-punishment intentions are aggregated into the decision matrix of group reward-punishment intentions $\tilde{L}={\left[{\tilde{L}}_{j}(\tilde{p})\right]}_{1\times m}$ which is shown in S4 Table.

#### Step 3

Measure the trend change level.

By Formula (23), we can calculate the degree of preference for rewards or punishments corresponding to each linguistic term in the evaluation of group reward-punishment intentions. Based on Formulas (24)–(25), we can calculate the probability gain level of each linguistic term in the evaluations of alternatives. Then the positive incentive points and the negative incentive points of probability gain level with different group reward-punishment preferences are respectively calculated by employing Formulas (26)–(27). Due to space limitation, we only list the positive incentive points and the negative incentive points of probability gain level for attribute *x*_1_ in period *t*_2_ in S5 Table. The total incentives of probability gain level with different group reward-punishment preferences are respectively calculated by employing Formulas (28)–(32), where only the total incentives of probability gain level for attribute *x*_1_ in different periods are shown in S6 Table. Based on Formula (33), we can calculate the incentive coefficient of trend change level with different group reward-punishment preferences. Due to space limitation, only the incentive coefficient of trend change level for attribute *x*_1_ in different periods is shown in S7 Table.

#### Step 4

Measure the trend change stability.

Based on Formulas (34)–(35), we can calculate probability gain stability of each linguistic term in the evaluations of alternatives. The positive incentive points and the negative incentive points of probability gain stability with different group reward-punishment preferences are respectively calculated by employing Formula (36). Due to space limitation, we only list the positive incentive points and the negative incentive points of probability gain stability for attribute *x*_1_ in S8 Table. Then the total incentives of probability gain stability with different group reward-punishment preferences respectively are calculated by employing Formulas (37)–(39), where only the total incentives of probability gain stability for attribute *x*_1_ are shown in S9 Table. Based on Formula (40), we can calculate the incentive coefficient of trend change stability with different group reward-punishment preferences. Due to space limitation, only the incentive coefficient of trend change stability for attribute *x*_1_ is shown in S10 Table.

#### Step 5

Aggregate the probabilistic linguistic group evaluations of different periods.

Based on Formula (41), we can calculate the composite incentive coefficient of trend change. By employing Formula (43), the probabilistic linguistic group evaluations of alternatives in all periods are aggregated into a dynamic group decision matrix *L* = [*L*_*ij*_(*p*)]_*n*×*m*_ which is shown in Table 1.

**Table 1:** The dynamic group decision matrix.

**Alternatives**	**Attributes**	
	*x*_1_	*x*_2_
*o*_**1**_	$\left\{s_{0}(0.18),s_{-0.6}(0.41),s_{-1.2}(0.41)\right\}$	$\left\{s_{3}(0.99),s_{1.8}(0.01)\right\}$
*o*_**2**_	$\left\{s_{0}(0.89),s_{-0.6}(0.11)\right\}$	$\left\{s_{0.6}(0.33),s_{0}(0.67)\right\}$
*o*_**3**_	$\left\{s_{0}(0.86),s_{-0.6}(0.13),s_{-1.2}(0.01)\right\}$	$\left\{s_{0.6}(0.99),s_{0}(0.01)\right\}$
*o*_**4**_	$\left\{s_{0.6}(1.00)\right\}$	$\left\{s_{0.6}(0.77),s_{0}(0.23)\right\}$
*o*_**5**_	$\left\{s_{3}(0.34),s_{0.6}(0.23),s_{0}(0.32),s_{-0.6}(0.11)\right\}$	$\left\{s_{3}(0.97),s_{0.6}(0.03)\right\}$
**Alternatives**	**Attributes**	
	*x*_**3**_	*x*_**4**_
*o*_**1**_	$\left\{s_{3}(0.33),s_{1.2}(0.38),s_{0.6}(0.29)\right\}$	$\left\{s_{3}(0.98),s_{1.2}(0.01),s_{0}(0.01)\right\}$
*o*_**2**_	$\left\{s_{3}(0.41),s_{1.2}(0.03),s_{0.6}(0.52),s_{0}(0.04)\right\}$	$\left\{s_{1.2}(0.80),s_{0.6}(0.19),s_{0}(0.01)\right\}$
*o*_**3**_	$\left\{s_{3}(0.44),s_{0.6}(0.10),s_{0}(0.46)\right\}$	$\left\{s_{1.2}(0.06),s_{0.6}(0.83),s_{0}(0.11)\right\}$
*o*_**4**_	$\left\{s_{3}(0.67),s_{1.2}(0.23),s_{0.6}(0.10)\right\}$	$\left\{s_{0.6}(0.98),s_{0}(0.02)\right\}$
*o*_**5**_	$\left\{s_{3}(0.41),s_{1.2}(0.09),s_{0.6}(0.28),s_{0}(0.22)\right\}$	$\left\{s_{3}(0.92),s_{1.8}(0.01),s_{1.2}(0.03),s_{0.6}(0.04)\right\}$

#### Step 6

Determine the positive ideal solution and the negative ideal solution.

Based on Formulas (8)–(9), we can choose the positive ideal solution $L{\left(p\right)}^{+}=(L_{1}{\left(p\right)}^{+},L_{2}{\left(p\right)}^{+},L_{3}{\left(p\right)}^{+},L_{4}{\left(p\right)}^{+})$ and the negative ideal solution $L{\left(p\right)}^{-}=(L_{1}{\left(p\right)}^{-},$ $L_{2}{\left(p\right)}^{-},L_{3}{\left(p\right)}^{-},L_{4}{\left(p\right)}^{-})$ in the group decision making process, where $L_{j}{\left(p\right)}^{+}=\{{\left(L_{j}^{\left(l\right)}\right)}^{+}|l=1,2,\cdots ,\#L_{ij}\}=\{{\left(L_{j}^{\left(1\right)}\right)}^{+},{\left(L_{j}^{\left(2\right)}\right)}^{+},\cdots ,{\left(L_{j}^{\left(\#L_{ij}\right)}\right)}^{+}\}$ and $L_{j}{\left(p\right)}^{-}=\{{\left(L_{j}^{\left(l\right)}\right)}^{-}|l=1,2,\cdots ,\#L_{ij}\}=\{{\left(L_{j}^{\left(1\right)}\right)}^{-},{\left(L_{j}^{\left(2\right)}\right)}^{-},\cdots ,{\left(L_{j}^{\left(\#L_{ij}\right)}\right)}^{-}\}$.

Therefore, based on the Table 1, we obtain the positive ideal solution *L*(*p*)^+^ and the negative ideal solution *L*(*p*)^−^ as follows:

$$L{\left(p\right)}^{+}=(\left\{s_{1.02},s_{0.14},s_{0},s_{0}\},\{s_{2.97},s_{0.02}\},\{s_{2.01},s_{0.46},s_{0.31},s_{0}\},\{s_{2.94},s_{0.50},s_{0.04},s_{0.02}\right\}),$$

$$L{\left(p\right)}^{-}=(\left\{s_{0},s_{-0.25},s_{-0.50},s_{-0.07}\},\{s_{0.20},s_{0}\},\{s_{0.99},s_{0.04},s_{0},s_{0}\},\{s_{0.07},s_{0},s_{0},s_{0},s_{0}\right\}).$$

#### Step 7

Calculate the deviation degrees between each alternative and the positive ideal solution, and the deviation degree between each alternative and the negative ideal solution.

Based on Formulas (10)–(11), we can calculate the deviation degree $d(o_{i},L{\left(p\right)}^{+})$ and $d(o_{i},L{\left(p\right)}^{-})$ respectively as follows:

$d(o_{1},L{\left(p\right)}^{+})=1.3569,\;d(o_{2},L{\left(p\right)}^{+})=3.9291,\;d(o_{3},L{\left(p\right)}^{+})=4.0560,$

$d(o_{4},L{\left(p\right)}^{+})=3.4174,\;d(o_{5},L{\left(p\right)}^{+})=0.7734,\;d(o_{1},L{\left(p\right)}^{-})=3.6489,$

$d(o_{2},L{\left(p\right)}^{-})=0.9109,\;d(o_{3},L{\left(p\right)}^{-})=0.9522,\;d(o_{4},L{\left(p\right)}^{-})=1.3656,$

$d(o_{5},L{\left(p\right)}^{-})=4.0051$

Then, $d_{\min}(o_{i},L{\left(p\right)}^{+})=0.7734$ and $d_{\max}(o_{i},L{\left(p\right)}^{-})=4.0051$.

#### Step 8

Calculate the closeness coefficient to the ideal solutions.

Based on Formula (12), we can calculate the closeness coefficient *CI*(*o*_*i*_) as follows:

$CI(o_{1})=-0.8434,\;CI(o_{2})=-4.8529,\;CI(o_{3})=-5.0067,$

$CI(o_{4})=-4.0778$, $CI(o_{5})=0.0000$.

#### Step 9

Rank the sustainable suppliers according to *CI*(*o*_*i*_).

According to the ascending order of *CI*(*o*_*i*_)f, the ranking of the potential sustainable suppliers is *o*_5_≻*o*_1_≻*o*_4_≻*o*_2_≻*o*_3_≻. *o*_5_ is the most appropriate sustainable supplier.

### Sensitivity analysis

**Fig 4:**
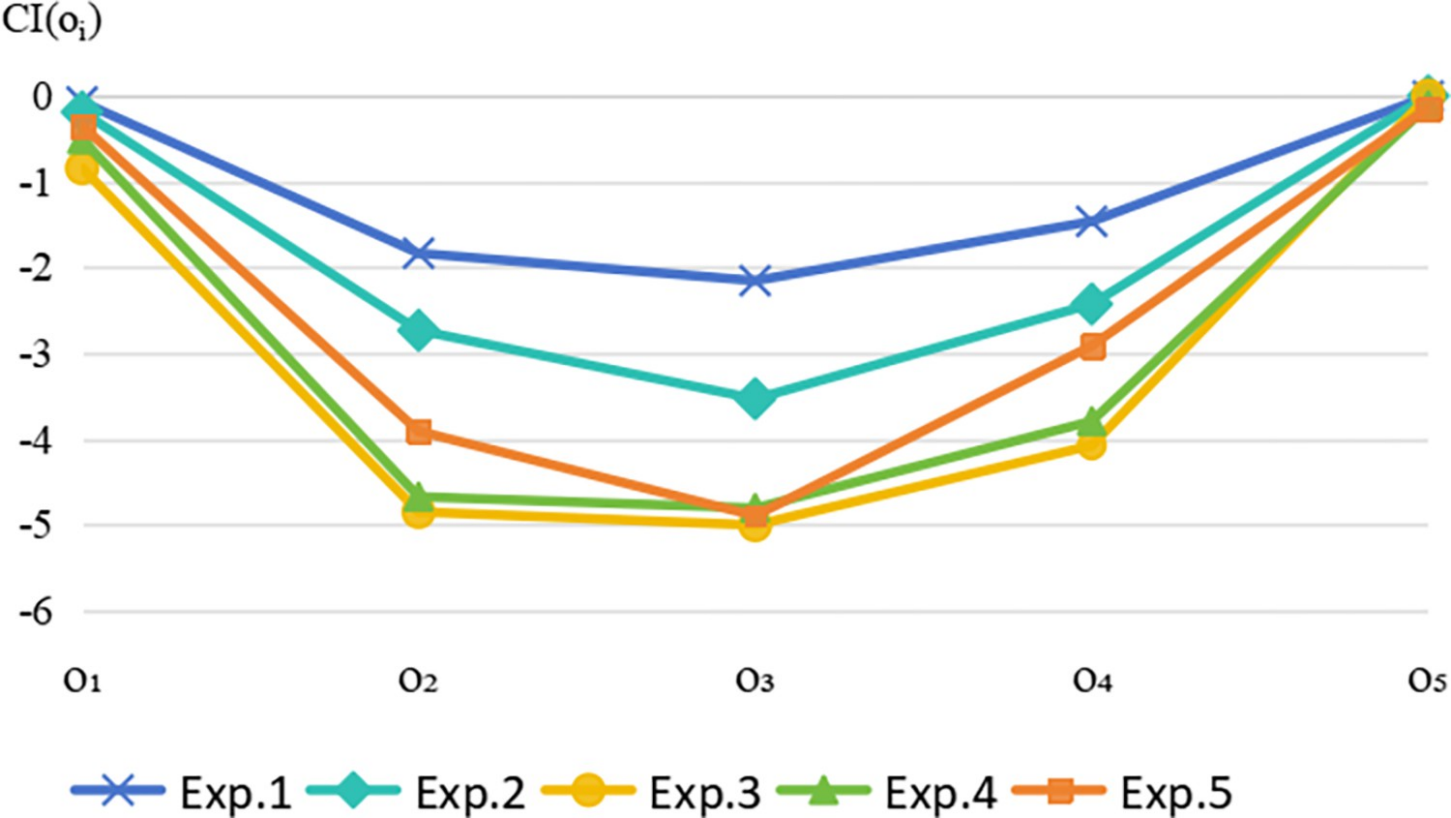
The closeness coefficients of sensitivity analysis.

To investigate the robustness of the proposed approach, we can implement the decision-making process of the aforementioned numerical example with several different sets of group reward-punishment intentions, as presented in Table 2. Obviously, Exp.3 represents the group reward-punishment intentions that place little focus on reward and punishment in the aforementioned numerical example, whereas other examples exhibit a preference for reward or punishment. Subsequently, the different closeness coefficients of sustainable suppliers with different group reward-punishment intentions are illustrated in Fig 4. As can be seen from Fig 4, the ranking of sustainable suppliers remains unchanged. Therefore, the impact of group reward-punishment intentions on the ranking results is relatively stable, indicating that the proposed method possesses robustness.

**Table 2:** The group reward-punishment intentions in the sensitivity analysis.

Example	The group reward-punishment intentions
**Exp.1**	$\left\{{\tilde{s}}_{1.8}(0.16),{\tilde{s}}_{1.2}(0.68),{\tilde{s}}_{0.6}(0.16)\right\}$
**Exp.2**	$\left\{{\tilde{s}}_{1.8}(0.04),{\tilde{s}}_{1.2}(0.32),{\tilde{s}}_{0.6}(0.64)\right\}$
**Exp.3**	$\left\{{\tilde{s}}_{1.2}(0.07),{\tilde{s}}_{0.6}(0.14),{\tilde{s}}_{0}(0.51),{\tilde{s}}_{-0.6}(0.28)\right\}$
**Exp.4**	$\left\{{\tilde{s}}_{-0.6}(0.75),{\tilde{s}}_{-1.2}(0.16),{\tilde{s}}_{-1.8}(0.09)\right\}$
**Exp.5**	$\left\{{\tilde{s}}_{-0.6}(0.35),{\tilde{s}}_{-1.2}(0.46),{\tilde{s}}_{-1.8}(0.19)\right\}$

### Comparative analysis

To further illustrate the effectiveness and feasibility of the proposed method, a comparison was made between the results obtained using the proposed method, the method based on hesitant fuzzy TOPSIS (HF-TOPSIS) [66] and the method based on probabilistic linguistic weighted geometric (PLWG) operator [16]. It should be noted that the HF-TOPSIS method operated within the hesitant fuzzy linguistic environment, where the probabilistic linguistic information employed in this study was transformed to hesitant fuzzy linguistic information. In contrast, the PLWG operator method utilized probabilistic linguistic information directly. Furthermore, for these methods, the weights of the periods calculated in this study were directly used. The ranking results of these different methods are presented in Table 3.

**Table 3:** The ranking results of different methods.

	the HF-TOPSIS method		the PLWG operator method		Proposed method	
	*CI*(*o*_*i*_)	Ranking results	*CI*(*o*_*i*_)	Ranking results	*CI*(*o*_*i*_)	Ranking results
*o*_**1**_	0.6057	1	0.0000	1	-0.8434	2
*o*_**2**_	0.4035	3	-2.5892	4	-4.8529	4
*o*_**3**_	0.2621	5	-2.9196	5	-5.0067	5
*o*_**4**_	0.3473	4	-2.3667	3	-4.0778	3
*o*_**5**_	0.4505	2	-0.0153	2	0.0000	1

According to Table 3, the ranking results of the HF-TOPSIS method is similar to those of the PLWA operator method, with *o*_1_ ranking first and *o*_5_ ranking second. However, it is worth noting that the ranking results of the above two methods are significantly different from those of the proposed method, such as the reverse order of *o*_1_ and *o*_5_. These inconsistent ranking results are mainly caused by three reasons: (1) In the HF-TOPSIS method, decision information is processed by HFLTS rather than by PTLS, which results in the incomplete expression of DMs’ opinions and preferences, thereby reducing the accuracy of the ranking results. (2) The HF-TOPSIS method only calculates the weight of DMs based on the consistency of their evaluations, and the PLWA operator method assigns equal weights to all DMs for different attributes. Neither method considers decision makers’ hesitant attitudes, differences in attribute cognition, and variability. This lack of consideration can negatively impact the accuracy of the ranking results. (3) Compared with the methods proposed in this study, the HF-TOPSIS method and the PLWA operator method only focus on the development state of alternatives, without considering their performance in terms of development trend. As a result, their one-sided decision-making processes lead to inaccurate ranking results.

Based on the aforementioned analysis, several advantages of utilizing the proposed method to rank sustainable suppliers can be summarized as follows.

1. Introduction of PLTS: The proposed method incorporates probabilistic linguistic term sets (PLTS) to evaluate sustainable suppliers. This enables a more effective handling of uncertainty faced by DMs and provides a more complete expression of their preferences.
2. Consideration of DMs’ hesitant attitude, attribute cognition difference and variability: The proposed method takes into account the hesitant attitudes of DMs, the differences and the variability in their attribute cognition. It calculates the weights of DMs based on their credibility for different attributes in different periods. This approach ensures a more effective and reliable reflection of the relative importance of different DMs.
3. Incorporation of development trend information: On the basis of considering the development state of alternatives, the proposed method also takes into account the development trend of alternatives. It achieves this by employing reward and punishment incentives to capture the decision information related to development trend. Furthermore, the proposed PLTOI operator is used to aggregate both the decision information of development state and development trend. This comprehensive approach leads to more reasonable and well-rounded ranking results.

These advantages collectively contribute to a more robust and accurate ranking of sustainable suppliers using the proposed method.

### Managerial insights

The relationship between enterprises and sustainable suppliers has evolved into a long-term partnership characterized by risk sharing, information sharing, and benefit sharing, instead of a buying and selling relationship. The implications derived from the example analysis are as follows:

1. Importance of considering DMs’ weights. In the process of selecting sustainable suppliers, the weights assigned to DMs directly impact the accuracy of group decision results. Enterprises should consider the hesitancy in DMs’ evaluation, the differences and the variability in DMs’ attribute cognition to determine their relative importance. The proposed method effectively determines the weights of DMs for different attributes at different stages, leveraging the characteristics of PLTS. This enables enterprises to obtain more reliable and robust group decision evaluations.
2. Significance of considering development trend. By comparing the ranking results of other methods with the proposed method, it becomes evident that whether the development trend of alternatives is considered has a significant impact on the ranking results. The development trend of alternatives reflects their growth potential, which directly impacts the comprehensive benefits of the enterprises in the long run. The proposed method evaluates alternatives from both their development state and trend, enabling enterprises to choose sustainable suppliers for long-term cooperation and mutual development.
3. Implementation of reward and punishment incentives. The proposed method incorporates different reward and punishment incentives for different suppliers, and classifies suppliers based on these incentives. This classification allows enterprises to guide suppliers with reward incentives to make breakthroughs in relevant attributes, while also guiding suppliers with punishment incentives to make continuous improvements in those attributes.

## Conclusion

Considering the current actual situation and previous research, a new multi-stage group decision making approach based on the PLTOI operator for selecting sustainable suppliers has been developed in this paper. The main contributions of this method are:

1. Introduction of PLTS for evaluating sustainable suppliers: The method incorporates the use of probabilistic linguistic term sets (PLTS) to evaluate sustainable suppliers. PLTS can effectively handle the uncertainty and fuzziness in linguistic evaluation, providing DMs with greater freedom in expressing their evaluations.
2. Balanced and reliable calculation of DMs’ weight: The method allows for a more balanced and reliable calculation of DMs’ weights for different attributes at different stages. By combining the hesitancy degree and similarity degree, the method can embed both the uncertainty and consensus of the DMs into their respective weights, enhancing the accuracy of the decision-making process.
3. Consideration of development trend through incentives: The method explores the differences in the development trend of alternatives by incorporating reward and punishment incentives. This provides DMs with decision information for the development trend, enabling facilitate DMs to make decisions that align with their own intentions.
4. Proposal of a multi-stage multi-attribute group decision making method based on PLTOI operator: The paper presents a comprehensive approach based on PLTOL operator for sustainable supplier selection. The method considers the development state and development trend of alternatives for various attributes at different stages, making the decision-making results more comprehensive, accurate, and convincing.

While the focus of this paper is on the independent evaluations by multiple decision-makers, it acknowledges that in a realistic world situation, decision makers can benefit from communication and exchanging opinions to improve the quality of their evaluations. Therefore, handling communication among multiple decision makers in the context of sustainable supplier selection is identified as a key direction for future research.

## Supporting information

S1 Table The standardtized probabilistic linguistic evaluation matrices of alternatives by *c*_1_. (DOC)

S2 Table The probabilistic linguistic evaluation matrix of reward-punishment intentions by all DMs. (DOC)

S3 Table The probabilistic linguistic group evaluation matrix for attribute *x*_1_. (DOC)

S4 Table The probabilistic linguistic evaluation matrix of group reward-punishment intentions. (DOC)

S5 Table The positive incentive points and the negative incentive points of probability gain level for attribute *x*_1_ in period *t*_2_. (DOC)

S6 Table The total incentives of probability gain level for attribute *x*_1_. (DOC)

S7 Table The incentive coefficient of trend change level for attribute *x*_1_ in different periods. (DOC)

S8 Table The positive incentive points and the negative incentive points of probability gain stability for attribute *x*_1_. (DOC)

S9 Table The total incentives of probability gain stability for attribute *x*_1_. (DOC)

S10 Table The incentive coefficient of trend change stability for attribute *x*_1_. (DOC)

## References

[1] Hendiani S, Sharifi E, Bagherpour M, Ghannadpour SF. A multi-criteria sustainability assessment approach for energy systems using sustainability triple bottom line attributes and linguistic preferences. Environment, Development and Sustainability. 2020; 22(8): 7771–7805. 10.1007/s10668-019-00546-7

[2] Rajnish K, Roshan G, Elmirzaev S. Optimization of Meat and Poultry Farm Inventory Stock Using Data Analytics for Green Supply Chain Network. Discrete Dynamics in Nature and Society. 2022; 2022: 1–8. 10.1155/2022/8970549

[3] Dang TT, Nguyen NA T, Nguyen VTT, Dang LTH. A Two-Stage Multi-Criteria Supplier Selection Model for Sustainable Automotive Supply Chain under Uncertainty. Axioms. 2022; 11(5):228. 10.3390/axioms11050228

[4] Duc DA, Van LH, Yu VF, Chou YY, Hien NV, Chi NT, et al. A dynamic generalized fuzzy multi-criteria croup decision making approach for green supplier segmentation. Plos one. 2021; 16(1): e0245187. doi: 10.1371/journal.pone.0245187 33493184 PMC7833157

[5] Wu C, Gao J, Barnes D. Sustainable partner selection and order allocation for strategic items: an integrated multi-stage decision-making model. International Journal of Production Research. 2023; 61(4):1076–1100. 10.1080/00207543.2022.2025945

[6] Jiang ZW, Wei GW, Guo YF. Picture fuzzy MABAC method based on prospect theory for multiple attribute group decision making and its application to suppliers selection. Journal of Intelligent & Fuzzy Systems. 2022; 42(4): 3405–3415. 10.3233/JIFS-211359

[7] Ning B, Wei G, Lin R, Guo YF. A novel MADM technique based on extended power generalized Maclaurin symmetric mean operators under probabilistic dual hesitant fuzzy setting and its application to sustainable suppliers selection. Expert Systems with Applications. 2022; 204: 117419. 10.1016/j.eswa.2022.117419

[8] Zulqarnain RM, Siddique I, Ahmad S, Iampan A, Jovanov G, Vranje D, et al. Pythagorean fuzzy soft Einstein ordered weighted average operator in sustainable supplier selection problem. Mathematical Problems in Engineering. 2021; 2021: 1–16. 10.1155/2021/2559979

[9] Zadeh LA. Fuzzy sets. Information and control. 1965; 8(3): 338–353.

[10] Turksen IB. Interval valued fuzzy sets based on normal forms. Fuzzy sets and systems. 1986; 20: 191–210.

[11] Atanassov KT. Intuitionistic fuzzy sets. Fuzzy sets and systems. 1986; 20: 87–96.

[12] Torra V. Hesitant fuzzy sets. International journal of intelligent systems. 2010; 25(6): 529–539. 10.1002/int.20418

[13] Yager RR, Abbasov AM. Pythagorean membership grades, complex numbers, and decision making. International Journal of Intelligent Systems. 2013; 28(5): 436–452. 10.1002/int.21584.

[14] Zadeh LA, Abbasov AM. The concept of a linguistic variable and its application to approximate reasoning—I. Information sciences. 1975; 8: 199–249.

[15] Rodriguez RM, Martinez L, Herrera F. Hesitant fuzzy linguistic term sets for decision making. IEEE Transactions on fuzzy systems. 2011; 20(1): 109–119.

[16] Pang Q, Wang H, Xu ZS. Probabilistic linguistic term sets in multi-attribute group decision making. Information Sciences. 2016; 369: 128–143. 10.1016/j.ins.2016.06.021

[17] Gou XJ, Xu ZS. Novel basic operational laws for linguistic terms, hesitant fuzzy linguistic term sets and probabilistic linguistic term sets. Information Sciences. 2016; 372: 407–427. 10.1016/j.ins.2016.08.034

[18] Wu SW, Chen XF. The research for PLTS normalization method based on minimum entropy change and its application in MAGDM problem. Plos one. 2022; 17(5): e0268158. doi: 10.1371/journal.pone.0268158 35522688 PMC9075675

[19] Zhong S, Zhang J, He X. Sustainable supply chain partner selection and order allocation: A hybrid fuzzy PL-TODIM based MCGDM approach. Plos one. 2022; 17(9): e0271194. doi: 10.1371/journal.pone.0271194 36137146 PMC9499258

[20] Kong QY, Wu LP. Combinatorial design of the MAUT and PAMSSEM II methods for multiple attributes group decision making with probabilistic linguistic information. Soft Computing. 2023; 27(4): 2093–2108. 10.1007/s00500-022-07379-z

[21] Liu PD, Wang P, Pedrycz W. Consistency-and consensus-based group decision-making method with incomplete probabilistic linguistic preference relations. IEEE Transactions on Fuzzy Systems. 2020; 29(9): 2565–2579.

[22] Wang P, Liu PD, Chiclana F. Multi-stage consistency optimization algorithm for decision making with incomplete probabilistic linguistic preference relation. Information Sciences. 2021; 556: 361–388. 10.1016/j.ins.2020.10.004

[23] Liu PD, Dang R, Wang P, Wu XM. Unit consensus cost-based approach for group decision-making with incomplete probabilistic linguistic preference relations. Information Sciences. 2023; 624: 849–880. 10.1016/j.ins.2022.12.114

[24] Darko AP, Liang DC. Probabilistic linguistic WASPAS method for patients’ prioritization by developing prioritized Maclaurin symmetric mean aggregation operators. Applied Intelligence. 2022; 52(8): 1–19. 10.1007/s1048 9-021-02807-3

[25] Su WH, Luo DD, Zhang CH, Zeng SZ. Evaluation of online learning platforms based on probabilistic linguistic term sets with self-confidence multiple attribute group decision making method. Expert Systems with Applications. 2022; 208: 118153. 10.1016/j.eswa.2022.118153

[26] Wan XL, Teng ZW, Zhang Z, Liu XT, Du ZQ. Equity financing risk assessment based on PLTS-ER approach in marine ranching from the ecological and circular economy perspectives. Annals of Operations Research, 2023: 1–46. 10.1007/s10479-023-05222-8

[27] Grošelj P. Symmetric projection group approach for promoting homogeneity in the analytic hierarchy process. Computers & Operations Research. 2021; 133: 105343. 10.1016/j.cor.2021.105343

[28] Davoudabadi R, Mousavi SM, Mohagheghi V. A new last aggregation method of multi-attributes group decision making based on concepts of TODIM, WASPAS and TOPSIS under interval-valued intuitionistic fuzzy uncertainty. Knowledge and Information Systems. 2020; 62(4): 1371–1391. 10.1007/s10115-019-01390-x

[29] Mohagheghi V, Mousavi SM. D-WASPAS: addressing social cognition in uncertain decision-making with an application to a sustainable project portfolio problem. Cognitive Computation. 2020; 12(3): 619–641. 10.1007/s12559-019-09679-3

[30] Liu LM, Bin Z, Shi B. Sustainable supplier selection based on regret theory and QUALIFLEX method. International Journal of Computational Intelligence Systems.2020; 13(1): 1120–1133. 10.2991/ijcis.d.200730.001

[31] Meng FY, Pedrycz W, Tang J. Interactive algorithms for normalized probabilistic linguistic preference relations in view of the disjunctive probability based consistency and consensus analysis. Engineering Applications of Artificial Intelligence. 2021; 104: 104363. 10.1016/j.engappai.2021.104363

[32] Li C, Huang H, Luo Y. An Integrated Two-Dimension Linguistic Intuitionistic Fuzzy Decision-Making Approach for Unmanned Aerial Vehicle Supplier Selection. Sustainability. 2022; 14(18): 11666. 10.3390/su141811666

[33] Giannakis M, Dubey R, Vlachos I. Supplier sustainability performance evaluation using the analytic network process. Journal of cleaner production. 2020; 247: 119439. 10.1016/j.jclepro.2019.119439

[34] Sánchez-Garrido AJ, Navarro IJ, García J. An Adaptive ANP & ELECTRE IS-Based MCDM Model Using Quantitative Variables. Mathematics. 2022; 10(12): 2009. 10.3390/math10122009

[35] Mishra AR, Saha A, Rani P. Sustainable supplier selection using HF-DEA-FOCUM-MABAC technique: a case study in the Auto-making industry. Soft Computing. 2022; 26(17): 8821–8840. doi: 10.1007/s00500-022-07192-8 35677555 PMC9164192

[36] Nazari-Shirkouhi S, Tavakoli M, Govindan K. A hybrid approach using Z-number DEA model and Artificial Neural Network for Resilient supplier Selection. Expert Systems with Applications. 2023; 222: 119746. 10.1016/j.eswa.2023.119746

[37] Wang CN, Yang FC, Vo NTM, Nguyen VTT. Enhancing Lithium-Ion Battery Manufacturing Efficiency: A Comparative Analysis Using DEA Malmquist and Epsilon-Based Measures. Batteries. 2023; 9: 317. 10.3390/batteries9060317

[38] Abdullah L, Ong Z, Mohd MS. Single-valued neutrosophic DEMATEL for segregating types of criteria: a case of subcontractors’ selection. Journal of mathematics. 2021; 2021: 1–12. 10.1155/2021/6636029

[39] Giri BC, Molla MU, Biswas P. Pythagorean fuzzy DEMATEL method for supplier selection in sustainable supply chain management. Expert Systems with Applications. 2022; 193: 116396. 10.1016/j.eswa.2021.116396

[40] Mohapatra B, Tripathy S, Singhal D. A sustainable solution for lean barriers through a fuzzy DEMATEL methodology with a case study from the Indian manufacturing industry. International Journal of Lean Six Sigma. 2023; 14(4): 815–843. 10.1108/IJLSS-06-2022-0134

[41] Sun YH, Cai YL. A flexible decision-making method for green supplier selection integrating TOPSIS and GRA under the single-valued neutrosophic environment. IEEE Access. 2021; 9: 83025–83040. 10.1109/ACCESS.2021.3085772

[42] Cai M, Hong YY. Improved TOPSIS Method Considering Fuzziness and Randomness in Multi-Attribute Group Decision Making. Mathematics. 2021; 10: 4200. 10.3390/math10224200

[43] Zhang N, Zhou Q, Wei GW. Research on Green Supplier Selection Based on Hesitant Fuzzy Set and Extended LINMAP Method. International Journal of Fuzzy Systems. 2022; 24(7): 3057–3066. 10.1007/s40815-022-01250-x

[44] Salimian S, Mousavi SM, Antucheviciene J. An interval-valued intuitionistic fuzzy model based on extended VIKOR and MARCOS for sustainable supplier selection in organ transplantation networks for healthcare devices. Sustainability. 2022; 14(7); 3795. 10.3390/su14073795

[45] Xiao JM, Cai M, Gao Y. A VIKOR-Based Linguistic Multi-Attribute Group Decision-Making Model in a Quantum Decision Scenario. Mathematics. 2022; 10(13): 2236. 10.3390/math10132236

[46] Zhao PY, Ji SF, Xue YT. An integrated approach based on the decision-theoretic rough set for resilient-sustainable supplier selection and order allocation. Kybernetes. 2023; 52(3): 774–808. 10.1108/K-11-2020-0821

[47] Alavi B, Tavana M, Mina H. A dynamic decision support system for sustainable supplier selection in circular economy. Sustainable Production and Consumption. 2021; 27: 905–920. 10.1016/j.spc.2021.02.015

[48] Bai CG, Zhu QY, Sarkis J. Circular economy and circularity supplier selection: a fuzzy group decision approach. International Journal of Production Research. 2022; 1–24. 10.1080/00207543.2022.2037779

[49] Wei DM, Meng D, Rong Y. Fermatean Fuzzy Schweizer–Sklar operators and BWM-entropy-based combined compromise solution approach: an application to green supplier selection. Entropy. 2022; 24(6): 776. doi: 10.3390/e24060776 35741498 PMC9223001

[50] Sarwar M, Zafar F, Majeed IA, Selection of Suppliers in Industrial Manufacturing: A Fuzzy Rough PROMETHEE Approach. Mathematical Problems in Engineering. 2022; 2022. 10.1155/2022/6141225

[51] Hua Z, Jing XC. A generalized Shapley index-based interval-valued Pythagorean fuzzy PROMETHEE method for group decision-making. Soft Computing. 2023; 27(10): 6629–6652. 10.1007/s00500-023-07842-5

[52] Kilic HS, Yalcin AS. Modified two-phase fuzzy goal programming integrated with IF-TOPSIS for green supplier selection. Applied Soft Computing. 2022; 93: 106371. 10.1016/j.asoc.2020.106371

[53] Khattak BK, Naseem A, Ullah M, Imran M, Ferik SE. Incorporating management opinion in green supplier selection model using quality function deployment and interactive fuzzy programming. Plos one. 2022; 17(6): e0268552. doi: 10.1371/journal.pone.0268552 35709147 PMC9202931

[54] Olanrewaju OG, Dong ZS, Hu S. Supplier selection decision making in disaster response. Computers & Industrial Engineering. 2020; 143: 106412. 10.1016/j.cie.2020.106412

[55] Kaur H, Singh SP. Multi-stage hybrid model for supplier selection and order allocation considering disruption risks and disruptive technologies. International Journal of Production Economics. 2021; 231: 107830. 10.1016/j.ijpe.2020.107830

[56] Guo SS, Gao Y, Guo J. A multi-stage group decision making for strategic supplier selection based on prospect theory with interval-valued q-rung orthopair fuzzy linguistic sets. Journal of Intelligent & Fuzzy Systems. 2021; 40(5): 9855–9871. 10.3233/JIFS-202415

[57] Li GX, Kou G, Li Y. A group decision making approach for supplier selection with multi-period fuzzy information and opinion interaction among decision makers. Journal of the Operational Research Society. 2022; 73: 855–868. 10.1080/01605682.2020.1869917

[58] Xie H, Ren Q, Duan W. New dynamic group DEMATEL decision-making method based on hesitant fuzzy linguistic term sets. International Journal of Fuzzy Systems. 2021; 23(7): 2118–2131. 10.1007/s40815-021-01081-2

[59] Hwang CL, Yoon K. Methods for multiple attribute decision making. Multiple attribute decision making: methods and applications a state-of-the-art survey. 1981; 58–191

[60] Mathew M, Chakrabortty RK, Ryan MJ. Selection of an optimal maintenance strategy under uncertain conditions: An interval type-2 fuzzy AHP-TOPSIS method. IEEE Transactions on Engineering Management. 2020; 69(4): 1121–1134. 10.1109/TEM.2020.2977141

[61] Jun HU, Junmin WU, Jie WU. TOPSIS hybrid multiattribute group decision-making based on interval pythagorean fuzzy numbers. Mathematical Problems in Engineering. 2022; 2021: 1–8. 10.1155/2021/5735272

[62] Zhang YX, Xu ZS, Liao HC. Water security evaluation based on the TODIM method with probabilistic linguistic term sets. Soft Computing. 2019; 23(15): 6215–6230. 10.1007/s00500-018-3276-9.

[63] Yi PT, Zhou Y, Guo YJ. A dynamic comprehensive evaluation method embodying development tendency. Operations Research and Management Science. 2016; 25: 175–180.

[64] Wang RT, Li XM, Li CT. Optimal selection of sustainable battery supplier for battery swapping station based on Triangular fuzzy entropy-MULTIMOORA method. Journal of Energy Storage. 2021; 34: 102013. 10.1016/j.est.2020.102013

[65] Wang H. Sustainable circular supplier selection in the power battery industry using a linguistic T-spherical fuzzy MAGDM model based on the improved ARAS method. Sustainability. 2022; 14(13): 7816. 10.3390/su14137816

[66] Liang R, Li R, Yan X, Xun ZZ, Wei X. Evaluating and selecting the supplier in prefabricated megaprojects using extended fuzzy TOPSIS under hesitant environment: A case study from China. Engineering, Construction and Architectural Management. 2023; 30(5): 1902–1931. 10.1108/ECAM-09-2021-0793

