# Supplementary material for: A multi-stage group decision making approach for sustainable supplier selection based on probabilistic linguistic time-ordered incentive operator
Source: PLoS One. 2023 Oct 31;18(10):e0293019. doi: 10.1371/journal.pone.0293019 (PMC10617744; doi:10.1371/journal.pone.0293019)
Supplement: S1 Table — (DOC) [file pone.0293019.s001.doc]

**S1 Table. The standardized probabilistic linguistic evaluation matrices of alternatives by $c_{1}$.**

| **Periods** | **Alternatives** | **Attributes** | |
| --- | --- | --- | --- |
|  |  | $x_{1}$ | $x_{2}$ |
| $t_{1}$ | $o_{1}$ | $\left\{s_{0}\left(0.18\right),s_{-1}\left(0.64\right),s_{-2}\left(0.18\right)\right\}$ | $\left\{s_{3}\left(0.30\right),s_{2}\left(0.70\right)\right\}$ |
|  | $o_{2}$ | $\left\{s_{-1}\left(1\right)\right\}$ | $\left\{s_{1}\left(1\right)\right\}$ |
|  | $o_{3}$ | $\left\{s_{-2}\left(1\right)\right\}$ | $\left\{s_{2}\left(0.80\right),s_{1}\left(0.10\right),s_{-1}\left(0.10\right)\right\}$ |
|  | $o_{4}$ | $\left\{s_{-1}\left(0.60\right),s_{-2}\left(0.40\right)\right\}$ | $\left\{s_{1}\left(1\right)\right\}$ |
|  | $o_{5}$ | $\left\{s_{1}\left(0.10\right),s_{0}\left(0.90\right)\right\}$ | $\left\{s_{2}\left(0.75\right),s_{1}\left(0.25\right)\right\}$ |
| $t_{2}$ | $o_{1}$ | $\left\{s_{1}\left(0.20\right),s_{0}\left(0.20\right),s_{-1}\left(0.60\right)\right\}$ | $\left\{s_{1}\left(0.75\right),s_{0}\left(0.25\right)\right\}$ |
|  | $o_{2}$ | $\left\{s_{1}\left(0.70\right),s_{0}\left(0.30\right)\right\}$ | $\left\{s_{1}\left(0.7\right),s_{0}\left(0.3\right)\right\}$ |
|  | $o_{3}$ | $\left\{s_{0}\left(0.25\right),s_{-1}\left(0.75\right)\right\}$ | $\left\{s_{2}\left(0.80\right),s_{1}\left(0.10\right),s_{-1}\left(0.10\right)\right\}$ |
|  | $o_{4}$ | $\left\{s_{1}\left(0.25\right),s_{0}\left(0.75\right)\right\}$ | $\left\{s_{1}\left(0.60\right),s_{0}\left(0.20\right),s_{-1}\left(0.20\right)\right\}$ |
|  | $o_{5}$ | $\left\{s_{0}\left(0.30\right),s_{-1}\left(0.70\right)\right\}$ | $\left\{s_{1}\left(0.60\right),s_{0}\left(0.40\right)\right\}$ |
| $t_{3}$ | $o_{1}$ | $\left\{s_{-1}\left(0.80\right),s_{-2}\left(0.20\right)\right\}$ | $\left\{s_{2}\left(0.60\right),s_{1}\left(0.20\right),s_{0}\left(0.20\right)\right\}$ |
|  | $o_{2}$ | $\left\{s_{-2}\left(0.60\right)\right\}$ | $\left\{s_{1}\left(0.80\right),s_{-1}\left(0.20\right)\right\}$ |
|  | $o_{3}$ | $\left\{s_{0}\left(0.20\right),s_{-1}\left(0.80\right)\right\}$ | $\left\{s_{1}\left(1\right)\right\}$ |
|  | $o_{4}$ | $\left\{s_{0}\left(0.20\right),s_{-1}\left(0.40\right),s_{-2}\left(0.40\right)\right\}$ | $\left\{s_{1}\left(0.20\right),s_{0}\left(0.80\right)\right\}$ |
|  | $o_{5}$ | $\left\{s_{2}\left(0.80\right),s_{-1}\left(0.20\right)\right\}$ | $\left\{s_{2}\left(0.70\right),s_{1}\left(0.30\right)\right\}$ |
| $t_{4}$ | $o_{1}$ | $\left\{s_{0}\left(0.20\right),s_{-1}\left(0.80\right)\right\}$ | $\left\{s_{3}\left(0.20\right),s_{1}\left(0.60\right),s_{0}\left(0.20\right)\right\}$ |
|  | $o_{2}$ | $\left\{s_{1}\left(0.875\right),s_{-2}\left(0.125\right)\right\}$ | $\left\{s_{1}\left(0.70\right),s_{0}\left(0.30\right)\right\}$ |
|  | $o_{3}$ | $\left\{s_{1}\left(0.50\right),s_{-1}\left(0.50\right)\right\}$ | $\left\{s_{2}\left(0.80\right),s_{1}\left(0.20\right)\right\}$ |
|  | $o_{4}$ | $\left\{s_{1}\left(1\right)\right\}$ | $\left\{s_{2}\left(0.30\right),s_{1}\left(0.70\right)\right\}$ |
|  | $o_{5}$ | $\left\{s_{0}\left(0.30\right),s_{-1}\left(0.70\right)\right\}$ | $\left\{s_{2}\left(0.20\right),s_{1}\left(0.70\right),s_{0}\left(0.10\right)\right\}$ |
| **Periods** | **Alternatives** | **Attributes** | |
|  |  | $x_{3}$ | $x_{4}$ |
| $t_{1}$ | $o_{1}$ | $\left\{s_{2}\left(0.70\right),s_{1}\left(0.30\right)\right\}$ | $\left\{s_{1}\left(0.25\right),s_{0}\left(0.75\right)\right\}$ |
|  | $o_{2}$ | $\left\{s_{1}\left(0.10\right),s_{0}\left(0.80\right),s_{-1}\left(0.10\right)\right\}$ | $\left\{s_{-1}\left(1\right)\right\}$ |
|  | $o_{3}$ | $\left\{s_{1}\left(0.20\right),s_{0}\left(0.80\right)\right\}$ | $\left\{s_{1}\left(1\right)\right\}$ |
|  | $o_{4}$ | $\left\{s_{0}\left(1\right)\right\}$ | $\left\{s_{1}\left(0.70\right),s_{0}\left(0.30\right)\right\}$ |
|  | $o_{5}$ | $\left\{s_{1}\left(1\right)\right\}$ | $\left\{s_{0}\left(0.50\right),s_{-1}\left(0.50\right)\right\}$ |
| $t_{2}$ | $o_{1}$ | $\left\{s_{1}\left(0.75\right),s_{0}\left(0.25\right)\right\}$ | $\left\{s_{0}\left(0.30\right),s_{-1}\left(0.50\right),s_{-2}\left(0.20\right)\right\}$ |
|  | $o_{2}$ | $\left\{\left\{s_{1}\left(0.10\right),s_{0}\left(0.80\right)\right.,s_{-1}\left(0.10\right)\right\}$ | $\left\{s_{-2}\left(0.40\right),s_{-3}\left(0.60\right)\right\}$ |
|  | $o_{3}$ | $\left\{s_{1}\left(0.30\right),s_{0}\left(0.70\right)\right\}$ | $\left\{s_{-2}\left(0.50\right),s_{-3}\left(0.50\right)\right\}$ |
|  | $o_{4}$ | $\left\{s_{1}\left(0.20\right),s_{0}\left(0.60\right),s_{-1}\left(0.20\right)\right\}$ | $\left\{s_{0}\left(0.30\right),s_{-1}\left(0.50\right),s_{-2}\left(0.20\right)\right\}$ |
|  | $o_{5}$ | $\left\{s_{1}\left(0.80\right),s_{0}\left(0.20\right)\right\}$ | $\left\{s_{-1}\left(0.70\right),s_{-2}\left(0.30\right)\right\}$ |
| $t_{3}$ | $o_{1}$ | $\left\{s_{0}\left(0.80\right),s_{-1}\left(0.10\right),s_{-2}\left(0.10\right)\right\}$ | $\left\{s_{2}\left(0.625\right),s_{1}\left(0.375\right)\right\}$ |
|  | $o_{2}$ | $\left\{s_{0}\left(1\right)\right\}$ | $\left\{s_{1}\left(0.40\right),s_{0}\left(0.20\right),s_{-1}\left(0.40\right)\right\}$ |
|  | $o_{3}$ | $\left\{s_{0}\left(0.40\right),s_{-1}\left(0.60\right)\right\}$ | $\left\{s_{1}\left(0.30\right),s_{0}\left(0.50\right),s_{-1}\left(0.20\right)\right\}$ |
|  | $o_{4}$ | $\left\{s_{1}\left(0.70\right),s_{0}\left(0.30\right)\right\}$ | $\left\{s_{1}\left(0.20\right),s_{0}\left(0.50\right),s_{-1}\left(0.30\right)\right\}$ |
|  | $o_{5}$ | $\left\{s_{1}\left(0.70\right),s_{0}\left(0.30\right)\right\}$ | $\left\{s_{2}\left(0.30\right),s_{-1}\left(0.70\right)\right\}$ |
| $t_{4}$ | $o_{1}$ | $\left\{s_{2}\left(0.20\right),s_{1}\left(0.60\right),s_{0}\left(0.20\right)\right\}$ | $\left\{s_{3}\left(0.30\right),s_{2}\left(0.50\right),s_{1}\left(0.20\right)\right\}$ |
|  | $o_{2}$ | $\left\{s_{1}\left(0.20\right),s_{0}\left(0.80\right)\right\}$ | $\left\{s_{2}\left(0.70\right),s_{1}\left(0.30\right)\right\}$ |
|  | $o_{3}$ | $\left\{s_{1}\left(0.70\right),s_{0}\left(0.30\right)\right\}$ | $\left\{s_{1}\left(0.20\right),s_{0}\left(0.80\right)\right\}$ |
|  | $o_{4}$ | $\left\{s_{2}\left(0.30\right),s_{1}\left(0.70\right)\right\}$ | $\left\{s_{2}\left(0.30\right),s_{1}\left(0.70\right)\right\}$ |
|  | $o_{5}$ | $\left\{s_{2}\left(0.70\right),s_{1}\left(0.20\right),s_{0}\left(0.10\right)\right\}$ | $\left\{s_{3}\left(0.20\right),s_{2}\left(0.20\right),s_{1}\left(0.60\right)\right\}$ |
