# Supplementary material for: A multi-stage group decision making approach for sustainable supplier selection based on probabilistic linguistic time-ordered incentive operator
Source: PLoS One. 2023 Oct 31;18(10):e0293019. doi: 10.1371/journal.pone.0293019 (PMC10617744; doi:10.1371/journal.pone.0293019)
Supplement: S2 Table — (DOC) [file pone.0293019.s002.doc]

**S2 Table. The probabilistic linguistic evaluation matrix of reward-punishment intentions by all DMs.**

| **Decision makers** | **Reward-punishment intentions** |
| --- | --- |
| $c_{1}$ | $\left\{\tilde{s}_{2}\left(0.1\right),\tilde{s}_{0}\left(0.8\right),\tilde{s}_{-1}\left(0.1\right)\right\}$ |
| $c_{2}$ | $\left\{\tilde{s}_{0}\left(0.7\right),\tilde{s}_{-2}\left(0.3\right)\right\}$ |
| $c_{3}$ | $\left\{\tilde{s}_{1}\left(0.2\right),\tilde{s}_{0}\left(0.8\right)\right\}$ |
