# Supplementary material for: A multi-stage group decision making approach for sustainable supplier selection based on probabilistic linguistic time-ordered incentive operator
Source: PLoS One. 2023 Oct 31;18(10):e0293019. doi: 10.1371/journal.pone.0293019 (PMC10617744; doi:10.1371/journal.pone.0293019)
Supplement: S3 Table — (DOC) [file pone.0293019.s003.doc]

**S3 Table. The probabilistic linguistic group evaluation matrix for attribute $x_{1}$.**

| **Alternative**s | **Periods** | |
| --- | --- | --- |
|  | $t_{1}$ | $t_{2}$ |
| $o_{1}$ | $\left\{s_{-1.2}\left(0.74\right),s_{-1.8}\left(0.26\right)\right\}$ | $\left\{s_{0}\left(0.16\right),\text{ }s_{-0.6}\left(0.32\right),s_{-1.2}\left(0.49\right),s_{-1.8}\left(0.03\right)\right\}$ |
| $o_{2}$ | $\left\{s_{-1.2}\left(1\right)\right\}$ | $\left\{s_{0.6}\left(0.04\right),\text{ }s_{0}\left(0.69\right),s_{-0.6}\left(0.24\right),s_{-1.2}\left(0.03\right)\right\}$ |
| $o_{3}$ | $\left\{s_{-0.6}\left(0.01\right),s_{-1.2}\left(0.18\right),s_{-1.8}\left(0.81\right)\right\}$ | $\left\{s_{0}\left(0.08\right),s_{-0.6}\left(0.40\right),s_{-1.2}\left(0.52\right)\right\}$ |
| $o_{4}$ | $\left\{s_{-0.6}\left(0.03\right),s_{-1.2}\left(0.72\right),s_{-1.8}\left(0.25\right)\right\}$ | $\left\{s_{0.6}\left(0.16\right),s_{0}\left(0.62\right),s_{-0.6}\left(0.22\right)\right\}$ |
| $o_{5}$ | $\left\{s_{1.2}\left(0.51\right),s_{0.6}\left(0.30\right),s_{0}\left(0.12\right),s_{-0.6}\left(0.07\right)\right\}$ | $\left\{s_{1.2}\left(0.06\right),s_{0.6}\left(0.32\right),s_{0}\left(0.48\right),s_{-0.6}\left(0.14\right)\right\}$ |
| **Alternatives** | **Periods** | |
|  | $t_{3}$ | $t_{4}$ |
| $o_{1}$ | $\left\{s_{-0.6}\left(0.14\right),s_{-1.2}\left(0.41\right),s_{-1.8}\left(0.45\right)\right\}$ | $\left\{s_{0.6}\left(0.02\right),s_{0}\left(0.41\right),s_{-0.6}\left(0.19\right),s_{-1.2}\left(0.38\right)\right\}$ |
| $o_{2}$ | $\left\{s_{-0.6}\left(0.80\right),s_{-1.2}\left(0.20\right)\right\}$ | $\left\{s_{0.6}\left(0.18\right),s_{0}\left(0.70\right),s_{-0.6}\left(0.02\right),s_{-1.2}\left(0.10\right)\right\}$ |
| $o_{3}$ | $\left\{s_{-0.6}\left(0.16\right),s_{-1.2}\left(0.84\right)\right\}$ | $\left\{s_{0.6}\left(0.40\right),s_{0}\left(0.50\right),s_{-0.6}\left(0.03\right),s_{-1.2}\left(0.07\right)\right\}$ |
| $o_{4}$ | $\left\{s_{0}\left(0.13\right),\text{ }s_{-0.6}\left(0.24\right),s_{-1.2}\left(0.57\right),s_{-1.8}\left(0.06\right)\right\}$ | $\left\{s_{1.8}\left(0.60\right),s_{1.2}\left(0.40\right)\right\}$ |
| $o_{5}$ | $\left\{s_{1.8}\left(0.69\right),s_{1.2}\left(0.17\right),s_{0.6}\left(0.03\right),s_{0}\left(0.01\right)\right\}$ | $\left\{s_{0}\left(0.03\right),\text{ }s_{-0.6}\left(0.20\right),s_{-1.2}\left(0.77\right)\right\}$ |
