# Supplementary material for: A multi-stage group decision making approach for sustainable supplier selection based on probabilistic linguistic time-ordered incentive operator
Source: PLoS One. 2023 Oct 31;18(10):e0293019. doi: 10.1371/journal.pone.0293019 (PMC10617744; doi:10.1371/journal.pone.0293019)
Supplement: S4 Table — (DOC) [file pone.0293019.s004.doc]

**S4 Table. The probabilistic linguistic evaluation matrix of group reward-punishment intentions.**

| **Attributes** | **Group reward-punishment intentions** |
| --- | --- |
| $x_{1}$ | $\left\{\tilde{s}_{1.2}\left(0.07\right),\tilde{s}_{0.6}\left(0.14\right),\tilde{s}_{0}\left(0.51\right),\tilde{s}_{-0.6}\left(0.28\right)\right\}$ |
| $x_{2}$ | $\left\{\tilde{s}_{1.2}\left(0.08\right),\tilde{s}_{0.6}\left(0.14\right),\tilde{s}_{0}\left(0.51\right),\tilde{s}_{-0.6}\left(0.25\right),\tilde{s}_{-1.2}\left(0.02\right)\right\}$ |
| $x_{3}$ | $\left\{\tilde{s}_{1.2}\left(0.08\right),\tilde{s}_{0.6}\left(0.14\right),\tilde{s}_{0}\left(0.51\right),\tilde{s}_{-0.6}\left(0.25\right),\tilde{s}_{-1.2}\left(0.02\right)\right\}$ |
| $x_{4}$ | $\left\{\tilde{s}_{1.2}\left(0.07\right),\tilde{s}_{0.6}\left(0.14\right),\tilde{s}_{0}\left(0.51\right),\tilde{s}_{-0.6}\left(0.28\right)\right\}$ |
