# Supplementary material for: A multi-stage group decision making approach for sustainable supplier selection based on probabilistic linguistic time-ordered incentive operator
Source: PLoS One. 2023 Oct 31;18(10):e0293019. doi: 10.1371/journal.pone.0293019 (PMC10617744; doi:10.1371/journal.pone.0293019)
Supplement: S5 Table — (DOC) [file pone.0293019.s005.doc]

**S5 Table. The positive incentive points and the negative incentive points of probability gain level for attribute $x_{1}$ in period $t_{2}$.**

| **Performance** | **Group reward-punishment intentions** | | | | | | | |
| --- | --- | --- | --- | --- | --- | --- | --- | --- |
|  | $\tilde{s}_{1.2}$ | | $\tilde{s}_{0.6}$ | | $\tilde{s}_{0}$ | | $\tilde{s}_{-0.6}$ | |
|  | $d_{l,k}^{\tilde{l}+}$ | $d_{l,k}^{\tilde{l}-}$ | $d_{l,k}^{\tilde{l}+}$ | $d_{l,k}^{\tilde{l}-}$ | $d_{l,k}^{\tilde{l}+}$ | $d_{l,k}^{\tilde{l}-}$ | $d_{l,k}^{\tilde{l}+}$ | $d_{l,k}^{\tilde{l}-}$ |
| $s_{3}$ | 0.0000 | 0.0000 | 0.0000 | 0.0000 | 0.0000 | 0.0000 | 0.0000 | 0.0000 |
| $s_{1.8}$ | 0.0000 | 0.0000 | 0.0000 | 0.0000 | 0.0000 | 0.0000 | 0.0000 | 0.0000 |
| $s_{1.2}$ | -0.0630 | -0.3420 | -0.0540 | -0.3060 | -0.0450 | -0.2700 | -0.0360 | -0.2340 |
| $s_{0.6}$ | 0.0788 | 0.0132 | 0.0904 | 0.0176 | 0.1020 | 0.0220 | 0.1136 | 0.0264 |
| $s_{0}$ | 0.4744 | 0.1706 | 0.5052 | 0.2008 | 0.5360 | 0.2310 | 0.5668 | 0.2612 |
| $s_{-0.6}$ | 0.1904 | 0.3456 | 0.1732 | 0.3308 | 0.1560 | 0.3160 | 0.1388 | 0.3012 |
| $s_{-1.2}$ | -0.5150 | 0.1420 | -0.5800 | 0.0760 | -0.6450 | 0.0100 | -0.7100 | -0.0560 |
| $s_{-1.8}$ | -0.4236 | -0.0774 | -0.4788 | -0.1032 | -0.5340 | -0.1290 | -0.5892 | -0.1548 |
| $s_{-2.4}$ | 0.0000 | 0.0000 | 0.0000 | 0.0000 | 0.0000 | 0.0000 | 0.0000 | 0.0000 |
| $s_{-3}$ | 0.0000 | 0.0000 | 0.0000 | 0.0000 | 0.0000 | 0.0000 | 0.0000 | 0.0000 |
