# Supplementary material for: A multi-stage group decision making approach for sustainable supplier selection based on probabilistic linguistic time-ordered incentive operator
Source: PLoS One. 2023 Oct 31;18(10):e0293019. doi: 10.1371/journal.pone.0293019 (PMC10617744; doi:10.1371/journal.pone.0293019)
Supplement: S6 Table — (DOC) [file pone.0293019.s006.doc]

**S6 Table. The total incentives of probability gain level for attribute $x_{1}$.**

| **Alternatives** | **Periods** | | | | | | | | | | | |
| --- | --- | --- | --- | --- | --- | --- | --- | --- | --- | --- | --- | --- |
|  | $t_{2}$ | | | | $t_{3}$ | | | | $t_{4}$ | | | |
|  | $\tilde{s}_{1.2}$ | $\tilde{s}_{0.6}$ | $\tilde{s}_{0}$ | $\tilde{s}_{-0.6}$ | $\tilde{s}_{1.2}$ | $\tilde{s}_{0.6}$ | $\tilde{s}_{0}$ | $\tilde{s}_{-0.6}$ | $\tilde{s}_{1.2}$ | $\tilde{s}_{0.6}$ | $\tilde{s}_{0}$ | $\tilde{s}_{-0.6}$ |
| $o_{1}$ | 0.0677 | 0.0542 | 0.0384 | 0.0161 | 0.2254 | -0.0814 | -0.2268 | -0.3722 | 0.4070 | 0.4061 | 0.3060 | 0.2059 |
| $o_{2}$ | 0.4823 | 0.3470 | 0.2118 | 0.0766 | 0.0989 | -0.1524 | -0.2424 | -0.3319 | 0.1655 | 0.1786 | 0.1182 | 0.0578 |
| $o_{3}$ | 0.4990 | 0.2981 | 0.0972 | -0.1037 | 0.3254 | 0.0929 | 0.0216 | -0.0497 | 0.5628 | 0.5191 | 0.4020 | 0.2849 |
| $o_{4}$ | 0.3706 | 0.2746 | 0.1788 | 0.0830 | -0.0608 | -0.3134 | -0.3918 | -0.4702 | 1.3259 | 1.1554 | 0.9114 | 0.6653 |
| $o_{5}$ | -0.1967 | -0.2966 | -0.3978 | -0.5676 | 1.3634 | 0.9917 | 0.7812 | 0.5722 | -1.0631 | -1.3195 | -1.6494 | -1.9793 |
