# Supplementary material for: A multi-stage group decision making approach for sustainable supplier selection based on probabilistic linguistic time-ordered incentive operator
Source: PLoS One. 2023 Oct 31;18(10):e0293019. doi: 10.1371/journal.pone.0293019 (PMC10617744; doi:10.1371/journal.pone.0293019)
Supplement: S7 Table — (DOC) [file pone.0293019.s007.doc]

**S7 Table. The incentive coefficient of trend change level for attribute $x_{1}$ in different periods.**

| **Alternatives** | **Periods** | | | | | | | | | | | |
| --- | --- | --- | --- | --- | --- | --- | --- | --- | --- | --- | --- | --- |
|  | $t_{2}$ | | | | $t_{3}$ | | | | $t_{4}$ | | | |
|  | $\tilde{s}_{1.2}$ | $\tilde{s}_{0.6}$ | $\tilde{s}_{0}$ | $\tilde{s}_{-0.6}$ | $\tilde{s}_{1.2}$ | $\tilde{s}_{0.6}$ | $\tilde{s}_{0}$ | $\tilde{s}_{-0.6}$ | $\tilde{s}_{1.2}$ | $\tilde{s}_{0.6}$ | $\tilde{s}_{0}$ | $\tilde{s}_{-0.6}$ |
| $o_{1}$ | 1.0338 | 1.0271 | 1.0192 | 1.0080 | 1.1122 | 0.9593 | 0.8871 | 0.8160 | 1.2008 | 1.2003 | 1.1518 | 1.1026 |
|  | 1.2366 | 1.1718 | 1.1055 | 1.0383 | 1.0494 | 0.9239 | 0.8794 | 0.8355 | 1.0826 | 1.0890 | 1.0590 | 1.0289 |
|  | 1.2444 | 1.1479 | 1.0486 | 0.9482 | 1.1613 | 1.0464 | 1.0108 | 0.9752 | 1.2742 | 1.2539 | 1.1983 | 1.1415 |
|  | 1.1832 | 1.1364 | 1.0892 | 1.0415 | 0.9696 | 0.8446 | 0.8066 | 0.7692 | 1.5803 | 1.5210 | 1.4266 | 1.3209 |
| $o_{5}$ | 0.9020 | 0.8528 | 0.8037 | 0.7236 | 1.5926 | 1.4588 | 1.3719 | 1.2785 | 0.5134 | 0.4218 | 0.3224 | 0.2428 |
