# Supplementary material for: A multi-stage group decision making approach for sustainable supplier selection based on probabilistic linguistic time-ordered incentive operator
Source: PLoS One. 2023 Oct 31;18(10):e0293019. doi: 10.1371/journal.pone.0293019 (PMC10617744; doi:10.1371/journal.pone.0293019)
Supplement: S8 Table — (DOC) [file pone.0293019.s008.doc]

**S8 Table. The positive incentive points and the negative incentive points of probability gain stability for attribute $x_{1}$.**

| **Performance** | **Group reward-punishment intentions** | | | | | | | |
| --- | --- | --- | --- | --- | --- | --- | --- | --- |
|  | $\tilde{s}_{1.2}$ | | $\tilde{s}_{0.6}$ | | $\tilde{s}_{0}$ | | $\tilde{s}_{-0.6}$ | |
|  | $d_{l,k}^{\tilde{l}+}$ | $d_{l,k}^{\tilde{l}-}$ | $d_{l,k}^{\tilde{l}+}$ | $d_{l,k}^{\tilde{l}-}$ | $d_{l,k}^{\tilde{l}+}$ | $d_{l,k}^{\tilde{l}-}$ | $d_{l,k}^{\tilde{l}+}$ | $d_{l,k}^{\tilde{l}-}$ |
| $s_{3}$ | 0.9873 | 0.9309 | 0.9891 | 0.9382 | 0.9909 | 0.9455 | 0.9927 | 0.9527 |
| $s_{1.8}$ | 0.9068 | 0.6743 | 0.9201 | 0.7018 | 0.9334 | 0.7293 | 0.9468 | 0.7568 |
| $s_{1.2}$ | 0.9431 | 0.8225 | 0.9512 | 0.8362 | 0.9594 | 0.8500 | 0.9675 | 0.8637 |
| $s_{0.6}$ | 0.9154 | 0.8339 | 0.9259 | 0.8410 | 0.9363 | 0.8482 | 0.9468 | 0.8554 |
| $s_{0}$ | 0.7159 | 0.5958 | 0.7247 | 0.6092 | 0.7336 | 0.6225 | 0.7424 | 0.6359 |
| $s_{-0.6}$ | 0.7897 | 0.6405 | 0.7989 | 0.6579 | 0.8080 | 0.6753 | 0.8171 | 0.6927 |
| $s_{-1.2}$ | 0.7469 | 0.6152 | 0.7683 | 0.6249 | 0.7896 | 0.6345 | 0.8110 | 0.6441 |
| $s_{-1.8}$ | 0.8929 | 0.7310 | 0.9082 | 0.7476 | 0.9235 | 0.7642 | 0.9388 | 0.7807 |
| $s_{-2.4}$ | 1.0000 | 1.0000 | 1.0000 | 1.0000 | 1.0000 | 1.0000 | 1.0000 | 1.0000 |
| $s_{-3}$ | 1.0000 | 1.0000 | 1.0000 | 1.0000 | 1.0000 | 1.0000 | 1.0000 | 1.0000 |
