# Supplementary material for: A multi-stage group decision making approach for sustainable supplier selection based on probabilistic linguistic time-ordered incentive operator
Source: PLoS One. 2023 Oct 31;18(10):e0293019. doi: 10.1371/journal.pone.0293019 (PMC10617744; doi:10.1371/journal.pone.0293019)
Supplement: S9 Table — (DOC) [file pone.0293019.s009.doc]

**S9 Table. The total incentives of probability gain stability for attribute $x_{1}$.**

| **Alternatives** | **Group reward-punishment intentions** | | | |
| --- | --- | --- | --- | --- |
|  | $\tilde{s}_{1.2}$ | $\tilde{s}_{0.6}$ | $\tilde{s}_{0}$ | $\tilde{s}_{-0.6}$ |
| $o_{1}$ | 0.4152 | 0.3256 | 0.2441 | 0.1636 |
| $o_{2}$ | 0.1776 | 0.1083 | 0.0311 | -0.0479 |
| $o_{3}$ | 0.1415 | 0.0761 | 0.0106 | -0.0548 |
| $o_{4}$ | 0.0060 | -0.0283 | -0.0627 | -0.1111 |
| $o_{5}$ | 0.0254 | -0.0476 | -0.1206 | -0.1935 |
