# Supplementary material for: A multi-stage group decision making approach for sustainable supplier selection based on probabilistic linguistic time-ordered incentive operator
Source: PLoS One. 2023 Oct 31;18(10):e0293019. doi: 10.1371/journal.pone.0293019 (PMC10617744; doi:10.1371/journal.pone.0293019)
Supplement: S10 Table — (DOC) [file pone.0293019.s010.doc]

**S10 Table. The incentive coefficient of trend change stability for attribute $x_{1}$.**

| **Alternatives** | **Group reward-punishment intentions** | | | |
| --- | --- | --- | --- | --- |
|  | $\tilde{s}_{1.2}$ | $\tilde{s}_{0.6}$ | $\tilde{s}_{0}$ | $\tilde{s}_{-0.6}$ |
| $o_{1}$ | 1.2047 | 1.1614 | 1.1214 | 1.0816 |
| $o_{2}$ | 1.0886 | 1.0541 | 1.0155 | 0.9760 |
| $o_{3}$ | 1.0706 | 1.0380 | 1.0053 | 0.9726 |
| $o_{4}$ | 1.0030 | 0.9858 | 0.9687 | 0.9445 |
| $o_{5}$ | 1.0127 | 0.9762 | 0.9398 | 0.9035 |
